# Metagenomic insights into potential PET hydrolases from Antarctic soils and rhizospheres

**DOI:** 10.3389/fmicb.2026.1749101

**Published:** 2026-07-16

**Authors:** Valentín Berríos-Farías, Sergio Guajardo-Leiva, Jorge Gallardo-Cerda, Cristóbal Galbán-Malagón, Claudia Egas, Marco A. Molina-Montenegro, Eduardo Castro-Nallar

**Affiliations:** 1 Instituto de Ciencias Biológicas, Universidad de Talca, Talca, Chile; 2 Centro de Ecología Integrativa, Universidad de Talca, Talca, Chile; 3 Dirección de Investigación, Vicerrectoría Académica, Universidad de Talca, Talca, Chile; 4 Centro de Genómica, Ecología y Medio Ambiente (GEMA), Universidad Mayor, Santiago, Chile; 5 Institute of Environment, Florida International University, Miami, FL, United States; 6 Centro Tecnológico de Innovación en Envases LABEN-CHILE, Santiago, Chile; 7 Centro de Investigación en Recursos Naturales y Sustentabilidad (CIRENYS), Universidad Bernardo O’Higgins, Santiago, Chile; 8 Cape Horn International Center, Universidad de Magallanes, Puerto Williams, Chile

**Keywords:** Antarctic rhizosphere, cold-adapted enzymes, metagenomics, plastic biodegradation, polyester hydrolases, rhizosphere microbiome

## Abstract

Polyethylene terephthalate (PET) is a persistent synthetic polymer that is increasingly detected in terrestrial environments, where it influences soil microbial activity and carbon cycling. Microorganisms capable of hydrolyzing PET and related polyesters constitute a valuable enzymatic resource for developing low-temperature biocatalysts and for advancing the understanding of soil functional adaptation to plastic pollution. Here, we conducted a metagenomic analysis of soil and rhizosphere samples from the Antarctic vascular plants *Deschampsia antarctica* and *Colobanthus quitensis*, as sources of microbial enzymes with potential PET-hydrolytic activity. Hidden Markov Models constructed from experimentally validated PET hydrolases identified 152 putative PET hydrolases (pPETHs) spanning multiple protein families. Four candidates exhibited amino acid motifs characteristic of *Ideonella sakaiensis* PETase, including the conserved alpha/beta hydrolase fold and the Ser-His-Asp catalytic triad. One candidate from a *Duganella* genome also contained a tryptophan residue associated with efficient product release during PET hydrolysis. Molecular docking and molecular dynamics analyses revealed that candidates retain the core catalytic architecture of established PET hydrolases, while simultaneously displaying structural signatures of cold adaptation. These findings demonstrate the diversity of PET-hydrolase-like genes within Antarctic rhizosphere and soil microbiomes, broadening the current understanding of microbial enzymatic potential under cold, oligotrophic conditions. The identified sequences highlight the rhizosphere as a reservoir of functional diversity relevant to soil biotechnology, cold-adapted catalysis, and microbial strategies for transforming recalcitrant carbon substrates.

## 1 Introduction

In recent decades, plastic (synthetic carbon-based polymers) pollution has become a significant environmental issue worldwide, with petroleum-based polymers now widespread across terrestrial and aquatic ecosystems (Amaral-Zettler et al., 2020; Azevedo-Santos et al., 2021; MacLeod et al., 2021). Due to their long persistence and poor management, plastics accumulate in soils, freshwater, and marine environments, where they fragment into microplastics (Petersen and Hubbart, 2021). These particles are readily incorporated into food webs and can propagate across trophic levels, thereby amplifying ecological risks (Dulta et al., 2025). In terrestrial ecosystems, microplastics also act as conduits for chemical pollutants, including heavy metals, thereby enhancing their mobility and availability in the rhizosphere (Dulta et al., 2025; Fang et al., 2025). Microplastics also provide new substrates for soil microbes, potentially affecting their metabolism and enzyme activity, making the rhizosphere a key area for understanding their ecological impacts (Lai et al., 2025; Schöpfer et al., 2022).

Polyethylene terephthalate (PET) is a widely used petroleum-based polymer known for its flexibility and durability; however, its complex structure makes it resistant to natural degradation (Jambeck et al., 2015; Qiu et al., 2024). Traditional management methods include thermomechanical recycling, which degrades PET into lower-quality plastics, and chemical recycling that requires high energy to break ester bonds. Both face efficiency and scalability limits, which translate into recycling only a small part of global PET (Blázquez-Sánchez et al., 2022; Garcia and Robertson, 2017). In this context, microbial biodegradation has become an important complementary mitigation strategy. PET hydrolases, enzymes capable of breaking down the ester bonds in PET, serve as a natural mechanism for bacteria and fungi in the environment to transform this synthetic carbon polymer (Ahmed et al., 2025; Bertocchini and Arias, 2023; Yoshida et al., 2016). Most known PET hydrolases are thermophilic and require high temperatures, near PET’s glass transition temperature (~65 °C), for efficient activity (Kawai et al., 2019; Wei and Zimmermann, 2017). The discovery of *Ideonella sakaiensis* PETase, a mesophilic enzyme active at moderate temperatures, has expanded the repertoire of potential biocatalysts (Yoshida et al., 2016). However, naturally occurring cold-active PET hydrolases are still underexplored.

Marine environments of Antarctica have recently become a promising source of such enzymes (Blázquez-Sánchez et al., 2022). Although remote, Antarctic environments are not pristine, and contamination with microplastics has been detected in snow, soils, and aquatic systems (Illuminati et al., 2024; Pellegrino et al., 2025). These synthetic carbon polymers coincide with conditions such as low temperatures, freeze–thaw cycles, oligotrophy, and high UV radiation, exerting strong selective pressures on microbes, often leading to enzymes with structural flexibility and enhanced catalytic efficiency (Correa and Abreu, 2020; Ramasamy et al., 2023). Recent research has demonstrated that Antarctic marine bacteria produce polyester hydrolases capable of degrading aliphatic and aromatic polymers at moderate temperatures (Blázquez-Sánchez et al., 2022), confirming that marine cold ecosystems harbor biocatalysts for degrading pollutants. Despite these advances, plant-associated soils in Antarctica remain largely unexplored in this context.

Rhizospheres are microbial hotspots enriched by root exudates, characterized by steep physicochemical gradients and known for their frequent gene exchange, which supports dense, metabolically adaptable microbial communities (Guajardo-Leiva et al., 2022; Pellegrino et al., 2025). They are also areas where microplastics can accumulate, creating intense, localized, and selective pressures on microbial communities (Wang et al., 2024), which may promote the evolution of enzymes capable of degrading synthetic polymers by breaking down natural polyesters such as cutin and suberin. Consequently, investigating the diversity and activity of esterases and cutinases in extreme or nutrient-poor ecosystems can offer valuable insights into the adaptive potential of soil microbiomes and their possible use in biotechnology (Jadhav et al., 2017; Pellegrino et al., 2025; Saberi Riseh et al., 2024).

Here, we identify and characterize putative PET-hydrolase–like genes from Antarctic soils and rhizospheres of native vascular plants *Deschampsia antarctica* and *Colobanthus quitensis*. Curated Hidden Markov Models, built from experimentally validated PET hydrolases, were used on shotgun metagenomic data to find candidate sequences. These candidates were further characterized using genome binning, motif analysis, and structural modeling. This work represents the first metagenomic-scale assessment of PET hydrolase diversity in Antarctic plant-associated soils, broadening the known ecological range of these enzymes and emphasizing their potential biotechnological importance for cold-adapted microbial functions.

## 2 Methods

### 2.1 Sample collection, DNA extraction and sequencing information

Forty-nine samples were obtained during the Austral Summer (February) as part of the “LV Expedición Científica Antártica (ECA-55).” Rhizosphere soils of *Colobanthus quitensis* (Rhi_Cq) and *Deschampsia antarctica* (Rhi_Da), as well as bare soils, were collected from four sites: Devils Point, North Beach, Rotch Dome (Ritli Hill), and Rotch Dome (Amadok Point) at Byers Peninsula, Livingston Island, Antarctica. Bare soils lack plants, mosses, lichens, or organic matter within 1.5 meters of any plant or group of plants. The soil attached to plants was divided into two categories: the rhizosphere-surrounding soil (RSS), which was released by shaking the roots, and the rhizosphere, a tightly adhered layer (2–5 mm) surrounding the roots. For all samples, the top 5–15 cm soil layer was collected at nearly sea level. Fine particles (≤2 mm) were separated from pebbles and roots with a sterile 2 mm metal mesh to ensure homogeneity. These soil particles were stored at −80 °C in 50 mL sterile tubes until DNA extraction. Details about samples and bacterial community composition were previously described (Berríos-Farías et al., 2025), and sample metadata can be found in NCBI Bioproject database PRJNA746701. All samples were processed under biosafety level 1 conditions. Plant and soil samples were collected with the permission of the Chilean Antarctic Institute (INACH).

Total DNA from rhizosphere, RSS, and open soil samples was extracted from 0.5 to 1 g of soil using the QIAamp PowerFecal DNA Kit (Qiagen, Düsseldorf, Germany) following the manufacturer’s protocols. DNA was quantified using the Qubit DNA High-Sensitivity Assay (Thermo Fisher Scientific, formerly Invitrogen). Quality was assessed by spectrophotometry (A260: A280 ratio), and DNA integrity was determined by agarose gel electrophoresis. After DNA extraction, metagenomic sequencing libraries were pooled, quantified by qPCR, and sequenced to generate 151-cycle paired-end reads per fragment on the Illumina NovaSeq 6,000 platform. A total of 98 FASTQ files were generated across the 49 samples, yielding an average of 10.09 Gb of raw data per sample (range: 5.21 to 13.53 Gb). For the purposes of this study, the RSS compartment was treated as part of the bulk soil. All raw sequencing data have been deposited in the NCBI database under BioProject PRJNA746701.

### 2.2 Reconstruction of metagenome-assembled genomes (MAGs)

Metagenome-assembled genomes (MAGs) were generated following a previously published clustering-guided co-assembly protocol (Berríos-Farías et al., 2025), including read processing, sample clustering, co-assembly, multi-tool binning, and taxonomic classification with GTDB-Tk and its r202 database (Chaumeil et al., 2020). Bacterial community analysis was performed using the phyloseq and vegan R packages (Dixon, 2003; McMurdie and Holmes, 2013). To compare alpha-diversity estimates across sample groups, the Effective Number of Species was calculated as the exponent of the Shannon-Wiener index.

### 2.3 Phylogenetic tree inference from representative genomes

We inferred a phylogenetic tree for the MAGs to represent the phylogenetic diversity from which the pPETHs are inferred. We used PhyloPhlan in supermatrix mode (--accurate --diversity high) with its bacterial core genes database to identify conserved prokaryotic genes in the dereplicated MAG dataset (Asnicar et al., 2020). The resulting multiple sequence alignment file was used as input for the IQ-TREE software (Nguyen et al., 2015) (−m MFP -bb 1,000 -bnni) to generate a phylogenetic tree from the concatenated detected genes. The resulting phylogenetic tree was incorporated into the phyloseq object and agglomerated at the Family level.

### 2.4 Identification of PETase sequences on assembled genomes

To identify PET-degrading sequences within the set of bins or MAGs, all bins were used without genome dereplication to broaden the search space for potential PET hydrolases, including intraspecific sequence variation. A search strategy was employed by elaborating Hidden Markov Models (HMMs) using the HMMER3 software (Mistry et al., 2013); this method has proven effective in previous studies for discovering novel enzymes with hydrolytic activity against the PET polymer (Acosta et al., 2025; Erickson et al., 2022).

Eighty-six unique amino acid sequences from proteins with experimentally confirmed hydrolytic activity on PET were collected from the PAZy database in February 2024 (Buchholz et al., 2022). These sequences were aligned using the T-Coffee multiple sequence aligner (Notredame et al., 2000). The resulting multiple sequence alignment was used to construct an HMM profile with HMMER3, employing the hmmbuild command with --seed 123 and --amino options. The profile was then compressed using hmmpress for downstream applications.

To identify putative PETases, all coding sequences (CDS) and their corresponding amino acid sequences were predicted using Prodigal (Hyatt et al., 2010) with translation Table 11 (prokaryotic translation code). Any partial gene or protein sequence was discarded from the total set of 1,484 redundant MAGs. This final protein dataset was screened for PETases using the constructed HMM with the hmmscan command in HMMER3, utilizing the --seed 123 option. The sequence search results were filtered using an e-value threshold of 1 × 10^−20^ and a minimum search score of 100.

### 2.5 Functional annotation of predicted PETases and sequence similarity network analysis

To classify the protein families associated with the identified PETases, the InterPro web interface was used as a protein annotation tool (Hunter et al., 2009). The results for each protein were tabulated, and annotations from Pfam (Protein Families Database) with a predicted PETase protein and an e-value threshold of <1 × 10e-3 were retained. Additionally, we inferred a sequence similarity network using the amino acid sequences of the predicted potential PET hydrolases via the EFI-EST web platform (Zallot et al., 2019). We set a minimum sequence identity cutoff of 50% (with an e-value <1e-5) between two proteins as the criterion for establishing an edge between two nodes. Cytoscape software was used for network visualization (Cytoscape, version 3.7.2; Shannon et al., 2003).

### 2.6 Phylogenetic analysis of DLH domain-containing proteins

From the identified proteins with putative catalytic activity over PET, along with the 86 PET-hydrolase sequences retrieved from the PAZy database, by using the Interpro web interface search tool, we searched for the dienelactone hydrolase (DLH) domain, a common *α*/*β* hydrolase domain, which, in the case of known PET hydrolases, contains the catalytic triad Ser-Asp-His responsible for the PET hydrolysis (Alam et al., 2020). We retained all protein sequences with Pfam domain annotations corresponding to “Dienelactone hydrolase.” To resolve a phylogenetic tree of these sequences, we incorporated three hydrolytic sequences from the *Bacillus* genus to serve as a phylogenetic tree outgroup (AFQ59091, MDK2600047, MCY7886136) (Hydroxyisourate hydrolase [Bacillus spizizenii]—Protein—NCBI, n.d.; Hydroxyisourate hydrolase [Bacillus stercoris]—Protein—NCBI, n.d.; Yu et al., 2012). Additionally, three PET-hydrolyzing sequences discovered in Antarctic soils were included. In total, 28 protein sequences were compiled into a single FASTA file and submitted to the ClustalW web interface (Larkin et al., 2007). The resulting multiple alignment file from ClustalW was used to infer a phylogenetic tree analysis with IQ-TREE, employing the following parameters: -m MFP, −cmax 15, -B 1200, and -bnni. The phylogenetic tree was plotted and analyzed in R using the ape and ggtree packages (Paradis and Schliep, 2019; Yu et al., 2017).

### 2.7 Protein structure prediction of DLH domain-carrying sequences and structural alignments

To examine the structural protein diversity and catalytic motifs of the predicted putative PET hydrolases, we used AlphaFold2 protein structure inference models via the ColabFold web interface (Jumper et al., 2021; Mirdita et al., 2022). We examined the structural diversity of predicted putative PET hydrolases that carry the DLH domain. Protein structure visualization was performed using PyMOL (Schrödinger LLC, 2015). Additionally, the structural alignment of candidate PET-hydrolyzing sequences was performed using the PyMOL alignment tool with the *Is*PETase 6eqe protein structure model (Austin et al., 2018).

### 2.8 Molecular dynamics of PET hydrolase candidates

In order to gain insights into cold adaptation from these four DLH domain-containing proteins, we decided to perform residue fluctuation analysis (RMSF) from molecular Dynamics of the protein structures of these candidate enzymes. (Amber24) (Case et al., 2025). The PDB files of the proteins were prepared using the ff14SB AMBER force field within a simulation box with periodic boundary conditions. A semi-octahedral water capsule was added 14 Å away from the outermost protein residues using the SPC/E water model. Counterions of Na+ and Cl– were added to neutralize the systems, based on the net charge of the protein structures, using the ff14SB force field.

The resulting topology and coordinate files were used as starting points for a two-step energy minimization. The first step consisted of solvent minimization using the steepest descent method for 2,500 steps, followed by 2,500 steps of the conjugate gradient method, with a harmonic restraint force of 10 kcal/mol/å^2^ applied to the protein atoms. Subsequently, a second energy minimization was performed on the entire system without restraints, using the same algorithms, order, and number of cycles. Statistical system data was recorded every 100 steps. Long-range electrostatic interactions were calculated using the Particle Mesh Ewald (PME) method, and a cutoff of 8.0 Å was applied for non-bonded interactions.

Following energy minimization, the systems were equilibrated at 300K using a two-step protocol. First, the systems were heated from 0 to 300K under an NVT ensemble for 100 picoseconds (50,000 steps of 2 fs) using a Langevin thermostat with a collision frequency of 2.0 ps^−1^, applying a weak positional restraint of 2 kcal/mol/Å^2^ to the protein backbone atoms. Next, a volume-expanding equilibration was performed under an NPT ensemble without restraints for another 100 picoseconds at 1 atm, using a Monte Carlo barostat to maintain pressure while holding the temperature at 300 K. The SHAKE algorithm was applied to constrain all bonds involving hydrogen atoms, allowing for a 2 fs time step.

Finally, triplicated 100-nanosecond production runs (50 million steps of 2 fs each) was executed for each system using the GPU-accelerated pmemd.cuda engine, maintaining the temperature at 300 K and pressure at 1 atm utilizing the Langevin thermostat and Monte Carlo barostat under an NPT ensemble. Residue fluctuation along the molecular dynamics (RMSF) was calculated using the AMBER atomicfluc program for residue alpha carbons. RMSF values were plotted using the R ggplot2 package (Wickham, 2016).

### 2.9 Molecular docking and interaction map

To gain insights in the feasibility of catalysis from the predicted protein structural models of the DLH domain-containing proteins, we decided to perform molecular docking of these four structural candidates alongside 2PET (ethylene glycol terephthalate (3:2)), PubChem CID: 12361634 as ligand (Supplementary Figure S5). To this end, molecular dynamics frames were clustered (considering only protein atoms) using the k-means clustering method from the cpptraj software (Case et al., 2025). The centroid frames of the most populated cluster were then used as the starting point for the molecular docking procedure. Briefly, the PDB protein structure file representing the centroid of the most populated cluster was prepared alongside with the 2PET dimer using Dockprep with default parameters from Chimera tools (Pettersen et al., 2004). A grid of 20x20x20 angstroms was prepared encapsulating the Ser-His-Asp catalytic triad of the respective four structural models. Finally Autodock vina (Trott and Olson, 2010) was used to perform flexible ligand docking. The ligand pose with the highest docking score was chosen to measure relevant atomic distances between residues and ligand atoms in Pymol (Schrödinger LLC, 2015). Finally, a 2D interaction map was abstracted from the pdb coordinates previously exported from Pymol using the Discovery Studio Visualizer Protein-Ligand interaction tool (Dassault Systèmes BIOVIA, 2024).

## 3 Results

### 3.1 Community structure from metagenome assembled genomes (MAGs)

To characterize the bacterial community and the taxonomic context of the novel genes encoding PET-degrading enzymes, we estimated bacterial composition profiles across all samples using MAG coverage information. The taxonomic classification of the 1,484 MAGs showed that 98.32% were assigned at the family level and 83.73% at the genus level. The most common families included Burkholderiaceae (11.9%), Chitinophagaceae (8.4%), Sphingomonadaceae (7.3%), Gemmatimonadaceae (5.6%), and Rhodanobacteraceae (4.6%). Next, we examined whether bacterial community diversity differed between soil and rhizosphere compartments. Using UNIFRAC distances, we estimated beta diversity among soil and rhizospheric metagenomes from *Deschampsia Antarctica* (Rhi_Da), *Colobanthus quitensis*, and the combined rhizospheres of both plants (Rhi_DaCq). Community dispersion is illustrated in the RDA ordination biplot (Supplementary Figure S1A). PERMANOVA analysis revealed significant differences among groups (*p*-value = 0.002), with the sample compartment accounting for 12.7% of the total variation in the community (*R*^2^ = 0.1269). Finally, a Wilcoxon test comparing alpha diversity measures (based on Shannon indices) of soil and rhizosphere samples found significant differences only between the bacterial communities of *Deschampsia Antarctica* and *Colobanthus quitensis* (*p* = 0.0481), and between *Deschampsia Antarctica*’s rhizosphere and bulk soil communities (*p* = 0.0376) (Supplementary Figure S1B).

### 3.2 Phylogenomic analysis of the recovered genomes and bacterial distribution

**Figure 1:**
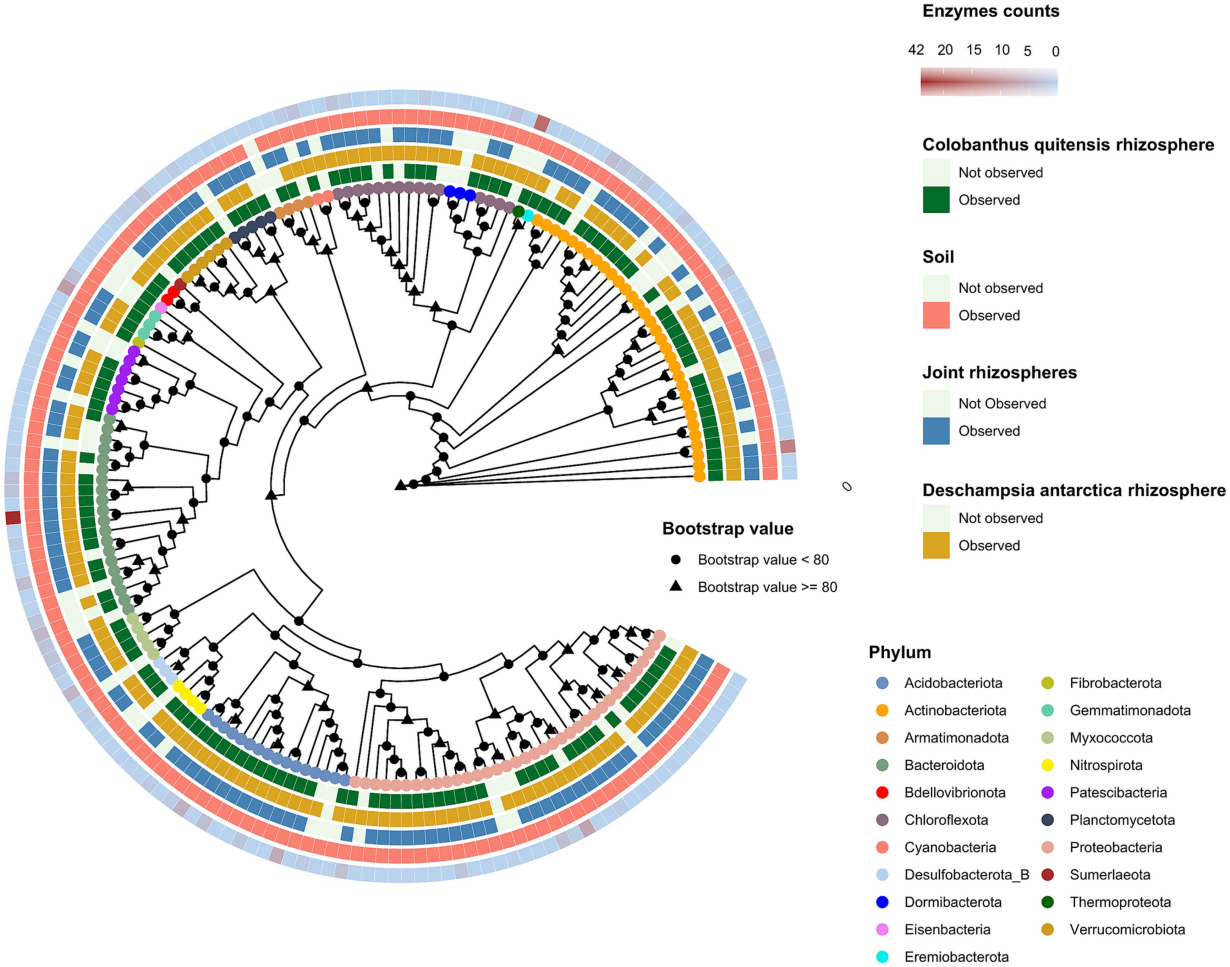
Phylogenetic distribution of soil and rhizosphere Antarctic bacterial genomes. Representatives of species genomes were selected to generate a phylogenomic tree depicting the bacterial communities in Antarctic soils and the rhizospheres of *the vascular plants Colobanthus quitensis* and *Deschampsia antarctica*. The tree was agglomerated at the Family taxonomic rank. Bootstrapping values are illustrated by the shapes of the tree nodes (triangles and circles). Tips represent bacterial families and are colored based on their phylum-level classification. The outer heatmap layers indicate whether a specific family is observed in the soil and rhizosphere compartments. Green, mustard, blue, and salmon heatmap rings represent a bacterial family in the rhizosphere of *Colobanthus quitensis (Rhi_*Cq*)*, *Deschampsia antarctica (Rhi_*Da*)*, the joint rhizosphere of both vascular plants (Rhi_DaCq), and soil, respectively. The outermost heatmap layer also displays the frequency of putative PET hydrolase sequences identified across bacterial families.

To strengthen the phylogenomic classification of MAGs, we analyzed the data using Phylophlan and IQ-TREE. The resulting phylogenomic tree was robust, with 79.5% of nodes exhibiting bootstrap values of 80% or higher, indicating high confidence in the tree topology. We grouped tree tips at the family taxonomic level (Figure 1) and observed that most bacterial phyla formed monophyletic clades. However, some clades included families from different phyla. Notably, a clade composed primarily of Chloroflexota genomes also included Dormibacterota.

Additionally, we identified a polyphyletic clade comprising seven families (based on GTDB taxonomy) from Gemmatimonadota, Bdellovibrionota, Eisenbacteria, and Sumerlaeota. We further examined the prevalence of bacterial families based on their MAG abundances, considering only those with trimmed mean abundances above zero. All detected bacterial families were present in the soil samples, except for the CAJBBX01 family, which was likely due to the stringent genome abundance estimation by coverM. In comparison, 77.5, 85.2, and 72.8% of all families were present in the Rhi_Cq, Rhi_Da, and Rhi_DaCq compartments, respectively.

### 3.3 Putative PET hydrolase sequences identified in Antarctic bacterial communities

**Figure 2:**
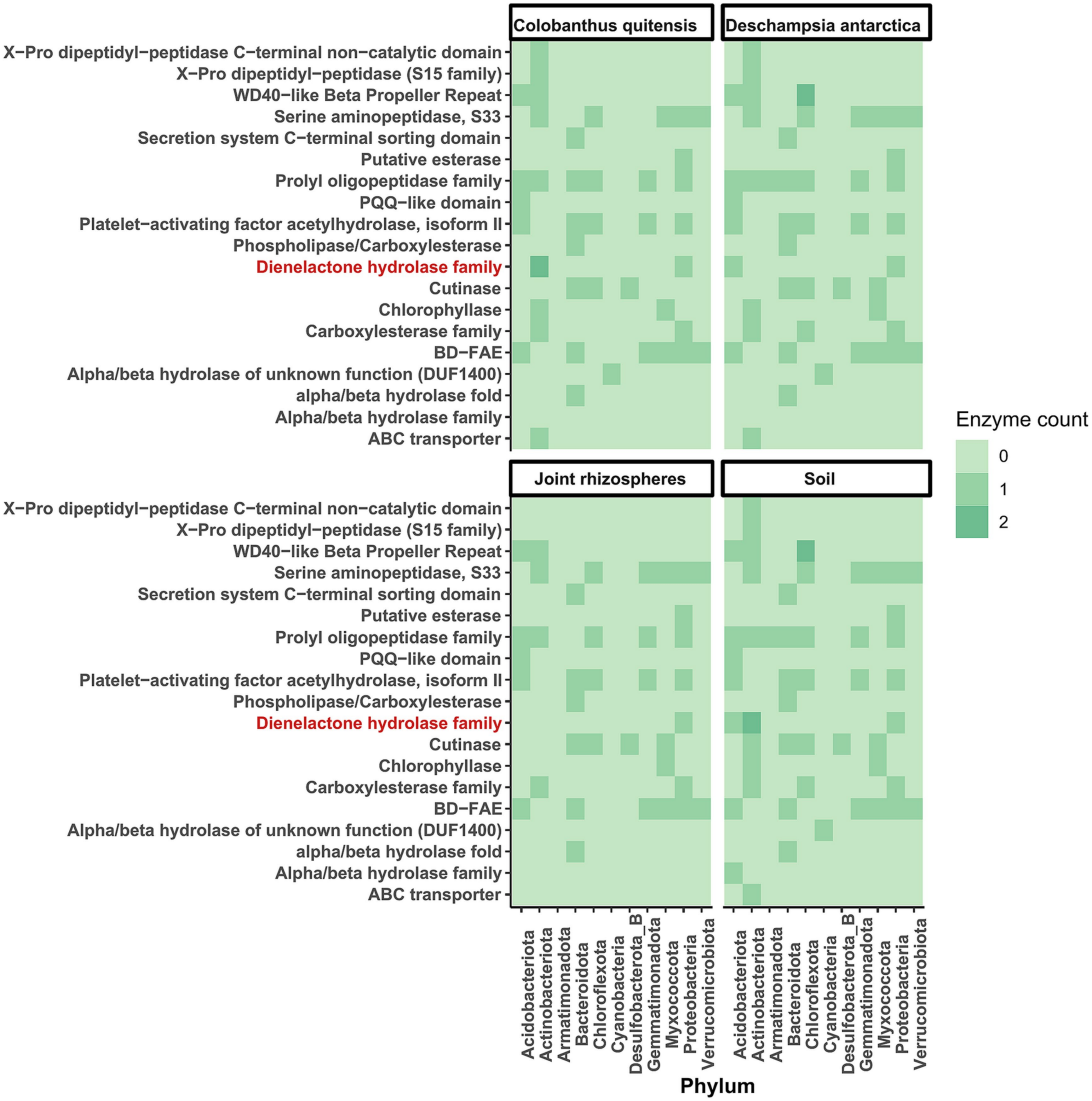
Protein annotation distribution across bacterial phyla and sample types. The 152 identified putative PET hydrolases were annotated with the Pfam database, and their distribution was mapped along bacterial Phyla and sample types. The *x*-axis delineates the bacterial phylum from which these sequences were identified, while the *y*-axis indicates the quantity of distinct protein families including Dienelactone hydrolases, cutinases, *α*/*β* hydrolases and the α/β hydrolases of unknown function derived from cyanobacteria (DUF1400) according to Pfam database annotations. The four grids relate to the distribution of these sequences across the soil and rhizospheric compartments of the vascular plants *Deschampsia antarctica* and *Colobanthus quitensis*. The enzyme’s distribution was determined by the presence or absence of a specific MAG in different compartments. This was assessed by checking if its estimated abundance value in a given compartment or sample type was greater than zero. In addition, enzymes annotated as “Dienelactone hydrolases” are highlighted in red color as this domain is found in *Ideonella sakaiensis* PETase (*Is*PETase) sequence and related PET hydrolases (Alam et al., 2020; Yoshida et al., 2016).

From 1,484 genomes, Prodigal identified 4,824,975 predicted proteins. Using hmmscan with our refined HMM, 28,741 protein sequences were identified as similar to those in the PAZy database. Rigorous filtering by *e*-value and HMM score yielded 152 putative PET hydrolase sequences. Bacteroidota contributed the highest number (42), followed by Actinobacteriota (26) and Chloroflexota (23) (Figure 1). Functional annotation using the Pfam database revealed that all 152 putative PET hydrolases had identifiable families: 30 were bifunctional feruloyl and acetyl xylan esterases (BD-FAE), 28 were prolyl oligopeptidases, and 21 were serine aminopeptidase S33. The set also included eight cutinases, two putative esterases, and four dienelactone hydrolases, the latter recognized as carrying a key catalytic domain for known PET hydrolases activity. These four were distributed across Actinobacteria (2), Proteobacteria (1), and Acidobacteria (1) (Figure 2 and Supplementary Table S2).

We also investigated the distribution of the 152 sequences among Antarctic soil and rhizosphere compartments. By linking these sequences to their respective bacterial host genomes, we evaluated their abundance in different sample types. Specifically, *α*/*β* hydrolases of unknown function (DUF1400) were present in all compartments except in the combined rhizospheres Rhi_DaCq. This pattern was also observed for the X-Pro dipeptidyl-peptidase C-terminal non-catalytic domain, X-Pro dipeptidyl-peptidase (S15 family), and ABC transporter protein families, all of which were absent from the combined vascular plants’ rhizospheres (Rhi_DaCq) (Figure 2). Additionally, cutinase sequences, known for hydrolyzing PET polymers, were found among diverse bacterial phyla and compartments.

### 3.4 Identified putative PET hydrolases sequence similarity network

**Figure 3:**
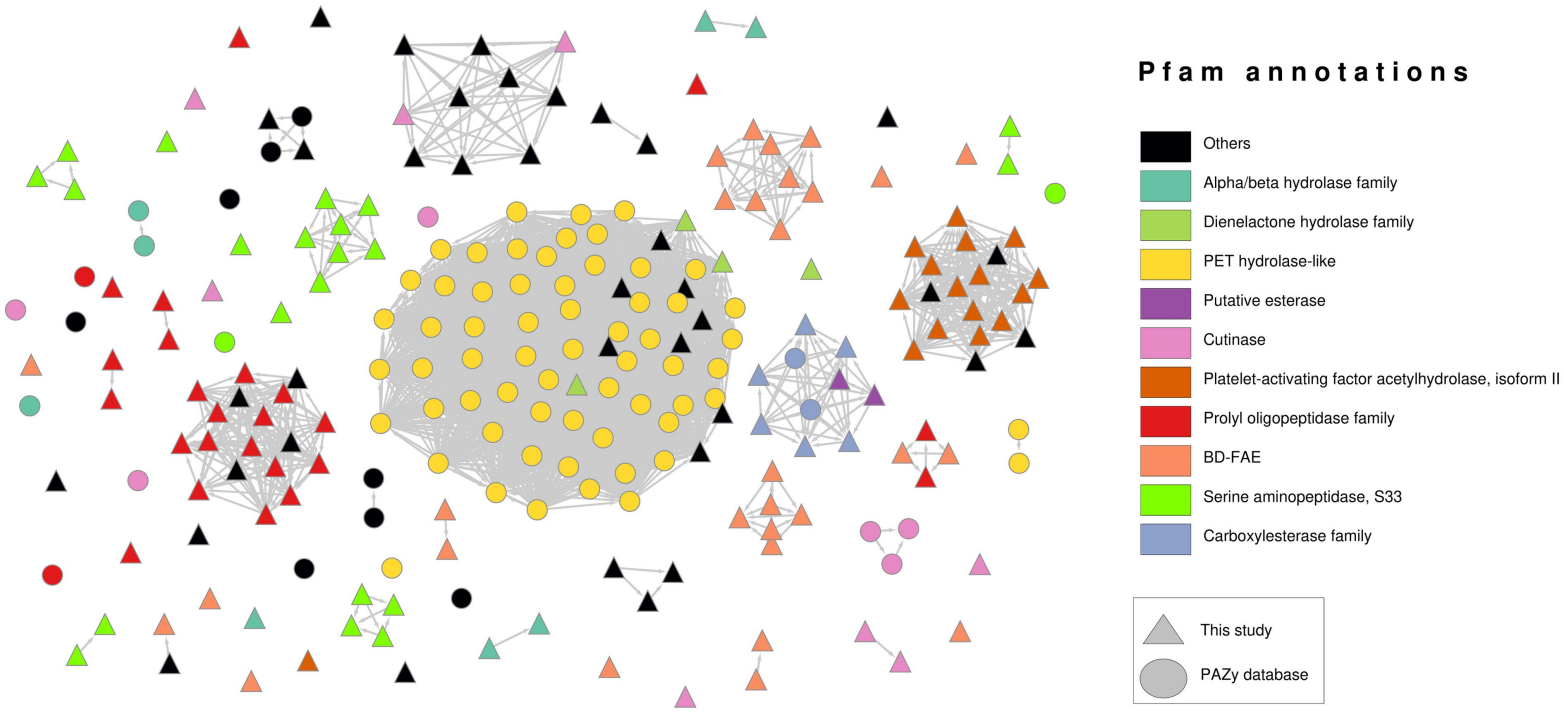
Sequence similarity network of identified putative PETases. The 152 putative PET hydrolases inferred in this study were subjected to Sequence Similarity Network (SSN) analysis, along with 86 experimentally verified PETases from the PAZy database. An edge between two nodes represents a sequence similarity of at least 50% between two proteins with an *e*-value < 1e^−5^. Node colors correspond to different protein families in the Pfam database. Circle nodes correspond to the PET hydrolases of the PAZy database, and triangle nodes correspond to the putative PET hydrolases inferred in this study.

To determine how closely the inferred putative PET-hydrolase sequences relate to known PET hydrolases, we performed a sequence similarity network (SSN) analysis comparing these proteins to 86 PET-hydrolyzing sequences from the PAZy database. The SSN, using a 50% amino acid sequence identity threshold, revealed several modules. The most populated module encompassed the majority of PET hydrolases from PAZy, all of which were annotated with the Pfam “PET hydrolase-like” terms. In this module, 11 proteins were identified in our study: three were labeled as dienelactone hydrolases (green triangles), and the remaining eight were grouped under the category “Others” (see Supplementary Table S3). In contrast, most of the putative PET hydrolases discovered in this work did not cluster within this main module. Although detected using an HMM trained on 86 verified PET hydrolases, these proteins share less than 50% sequence identity with PAZy hydrolases. Their Pfam annotations indicate a range of other protein families, including carboxyl esterases, putative esterases, serine aminopeptidases, and BD-FAEs (Figure 3).

### 3.5 Phylogenetic analysis of DLH-domain containing pPETHs

To assess the phylogenetic divergence of the inferred pPETHs relative to known PET hydrolase sequences, we searched for the DLH domain, which has previously been reported to be essential for PET polymer catalytic activity in bacterial cutinases such as *Is*PETase (Alam et al., 2020). Using Pfam annotations from the InterProScan results, we identified 22 sequences with the DLH domain. Four of these matched our predicted proteins and were present in MAGs classified as *Acidobacteriaceae*, *Duganella*, and *Pseudonocardiaceae*.

We designated these four DLH-domain candidates according to their host taxonomy: Acp1 (Acidobacteriaceae), Dup1 (Duganella), and Umep1 and Umep2 (both recovered from the same Umezawaea genome). These four identifiers correspond to the sequences flagged as “Candidate = Yes” in Supplementary Table S2 and to the highlighted rows in Figure 2, and are used consistently throughout the remainder of the manuscript. We also included three known PET-hydrolyzing sequences from Antarctic soils and three *Bacillus* DLH domain sequences without PET activity as outgroups. Multiple sequence alignment (MSA) revealed that all sequences shared the Ser-His-Asp catalytic triad (Supplementary Figure S2).

**Figure 4:**
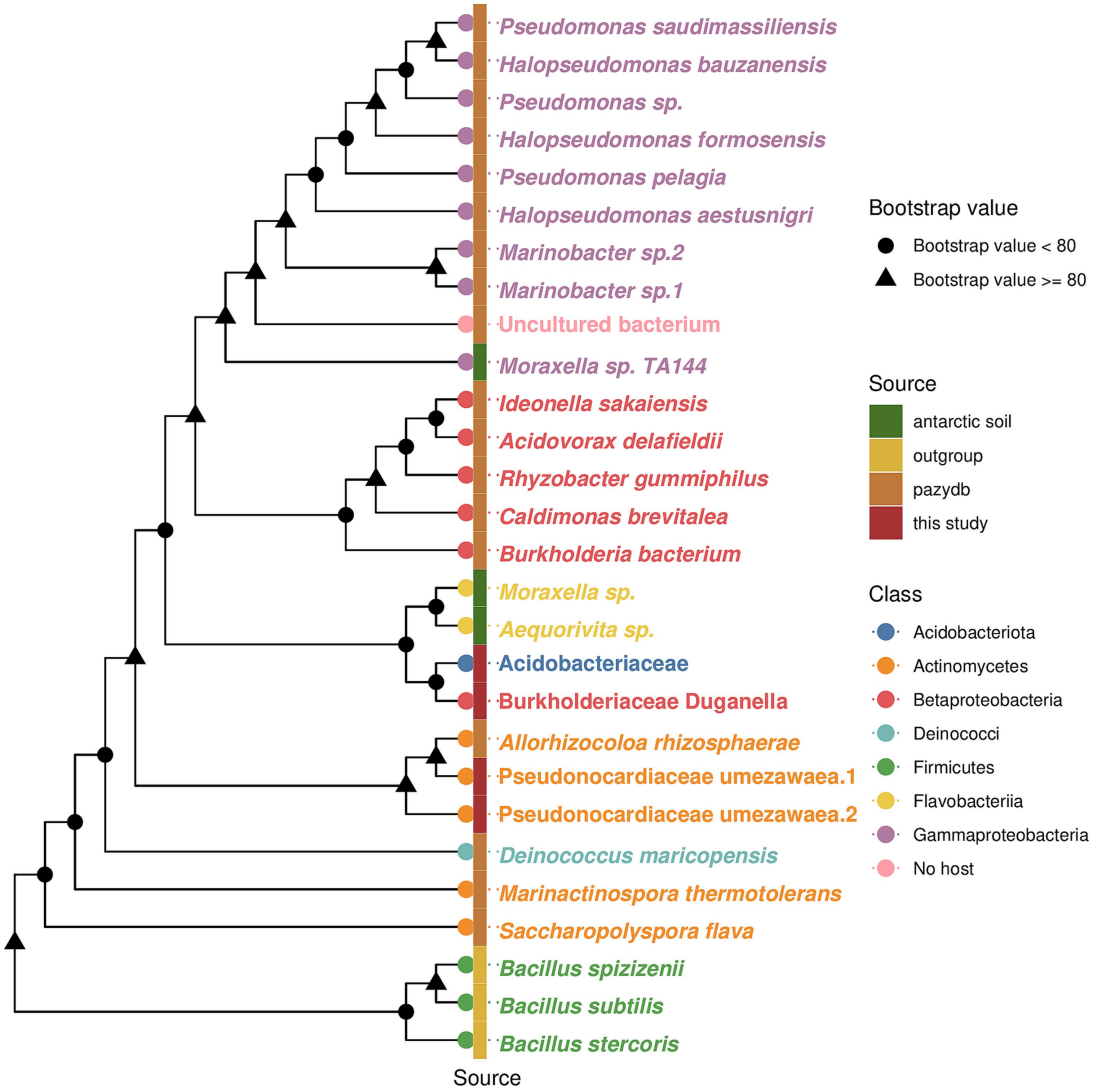
Rooted Phylogenetic tree of DLH Domain-Carrying PETase enzymes. The phylogenetic tree includes four putative PET hydrolases (pPETHs) identified in this study that possess the dienelactone hydrolase (DLH) domain, alongside known DLH domain-carrying PETases from the PAZy database. Three previously identified Antarctic soil-derived PETases were also incorporated. To root the tree, three *Bacillus* hydrolytic sequences with DLH domains but no reported PET degradation activity were included as outgroups. The heatmap column indicates the source of the sequence. Tree tip colors represent the bacterial Class of the species from which the proteins were extracted. Bacterial names are displayed next to the tree tips, and node shapes symbolize bootstrapping values.

Phylogenetic analysis showed that the major representatives were the classes *Gammaproteobacteria*, *Betaproteobacteria*, and *Actinobacteria* (Figure 4). Umep1 and Umep2, found within the same *Umezawaea* genome (Pseudonocardiaceae Family), clustered closely together, whereas Acp1 and Dup1 grouped in a clade with sequences from *Acidobacteriota* and *Betaproteobacteria* (Figure 4).

### 3.6 Structural and functional insights of DLH domain-containing putative PET hydrolases

**Figure 5:**
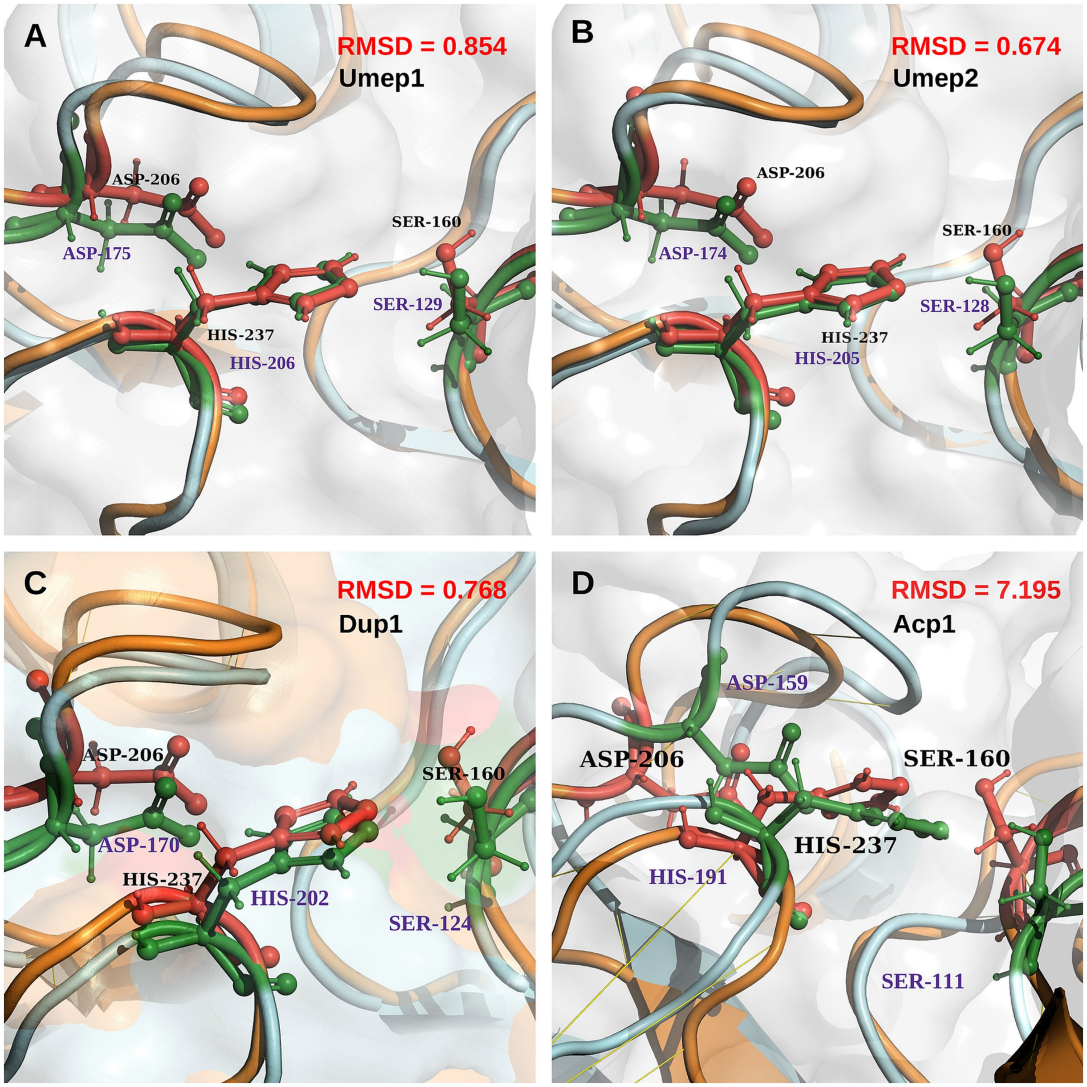
Overall protein-structure alignment of candidate PETases against *Is*PETase. AlphaFold2 structural models from the four identified pPETHs carrying the DLH domain were retrieved using the ColabFold web platform. Relaxed structures were aligned against the 6eqe PDB structure model of *Is*PETase. Orange-colored protein segments correspond to the *Is*PETase structure, while sky-blue segments are the four respective pPETHs. The four identified potential PET hydrolases, Umep1 **(A)**, Umep2 **(B)**, Dup1 **(C)**, and Acp1 **(D)**, possess the Ser-His-Asp catalytic triad characteristic of several PET-hydrolyzing enzymes. Serine, Histidine, and Aspartate residues of the 6eqe protein structure are labeled in black color, and the respective homolog catalytic residues names of the Acp1, Dup1, Umep1 and Umep2 residues are labeled in purple. Conversely, the catalytic triad residues in the four pPETHs are colored in green for each alignment. Root Mean Square Deviations (RMSD) of the pPETHs relative to the *Is*PETase 6eqe model are indicated in red in each panel.

To assess whether the sequence identity of the four pPETHs correlates with similar protein structures, we performed structural alignments against the *Is*PETase 6eqe PDB model. We predicted the structures of the four candidate pPETHs: Dup1, Umep1, Umep2, and Acp1. PyMOL alignments showed root-mean-square deviations (RMSDs) of 0.768, 0.854, 0.674, and 7.195 for Dup1, Umep1, Umep2, and Acp1, respectively (Figure 5). All four predicted structures showed Ser-Asp-His catalytic triad residues closely aligned with those of the 6eqe structure (Ser160, Asp206, and His237). Overall, the Acp1 protein had the highest RMSD value compared to the 6eqe model (Figure 5). Additionally, Molecular dynamics of the four protein structures were performed to assess residue fluctuations and potential psychrotolerance features. RMSF were calculated upon the 100 ns trajectories (Supplementary Figure S3). Mean Root Mean Square Fluctuation (RMSF) values varied across the four potential PET hydrolases (pPETHs) relative to the IsPETase 6eqe structure (0.543 Å; Supplementary Table S4). Umep1 exhibited a markedly higher mean RMSF (4.193 Å), while Acp1 (0.834 Å) and Dup1 (0.650 Å) were moderately higher than the reference. Umep2 showed the value closest to IsPETase (0.566 Å) (Supplementary Table S4). Finally, the residue fluctuations of Umep2 and Dup1 tends to be similar to the RMSF pattern of *Is*PETase residues, with the exception of Acp1 protein structure which possess higher RMSF values for residues surrounding the catalytic residues (Supplementary Figure S3), suggesting more flexibility around the catalytic groove which may be associated with cold adaptation.

Additionally, in the four pPETHs the catalytic His and Asp residues showed comparatively higher RMSF values than the catalytic Ser across the simulations. We report this as a descriptive observation of the simulation trajectories; its functional relevance, if any, would require experimental validation.

### 3.7 Molecular docking with 2PET dimer

To gain insights into enzyme functionality in PET polymer hydrolysis from the pPETHs, we performed a molecular docking analysis with flexible ligands. The centroid protein structure from the most populated cluster from the respective molecular dynamics simulations was used as a representative protein conformer. We chose the ligand pose with the most favorable docking score (most negative binding ΔG(kcal/mol)), which represents the protein:ligand conformation with the most favorable global contacts in the catalytic pocket according to the Vina empirical scoring function.

Free energy profiles of the best protein-ligand conformers ranged from −7.4 kcal/mol to −4.7 kcal/mol (Supplementary Table S6), with the Acp1 complex exhibiting the highest binding affinity and Dup1 the lowest. The Acp1 model also displayed the highest number of ligand-stabilizing contacts (Supplementary Table S7) with 10 interactions. Because hydrogen bonding is a primary driver for specific substrate positioning, it is noteworthy that hydrogen bond networks were observed across all five structures, including the *Is*PETase reference.

In the highly affinitive Acp1:2PET complex, seven hydrogen bonds tightly coordinate the substrate. Key among these are the hydrogen bonds forming the oxyanion hole, composed of Leu112 (distance: 1.83 Å) and Ser34 (distance: 2.31 Å), which stabilize the 2PET carbonyl group, a critical requirement for ligand stabilization (Burgin et al., 2024; Supplementary Figures S4A,E). Additionally, a hydrogen bond from the Ser111 beta carbon hydrogen to an oxygen carbonyl atom (O8) of 2PET is observed at a distance of 2.42 Å (Supplementary Table S7 and Supplementary Figure S5).

Following this primary hydrogen bond network, secondary hydrophobic interactions further stabilize the Acp1 conformer. Three hydrophobic interactions are observed: *π*-π stacked, π-π T-shaped, and π-alkyl. Specifically, π-π stacking interactions from Acp1 Phe115 and Tyr73 to one PET aromatic ring are present, mirroring the key stabilizing π-π stacked interactions previously described for *Is*PETase from Trp159 and Trp185 to one of the PET aromatic rings (Burgin et al., 2024).

Regarding the Umep1, Umep2, and Dup1 protein structures, these specific interactions described for the Acp1:2PET complex are not observed. Still, hydrogen bond interactions exist between the 2PET ligand and respective protein residues (Supplementary Table S7). Additionally, aromatic ring stabilization is observed with π-alkyl and π-π T-shaped interactions in the Umep1:2PET conformer with the help of Ala154 and Phe156, respectively (Supplementary Table S7). In the Umep2 conformer, only one π-π stacked interaction is observed to stabilize one aromatic ring of the 2PET ligand with the aid of Tyr153 (Supplementary Table S7). With the exception of the Acp1 protein structure, no interaction between the catalytic serine and the carbonyl oxygen of the 2PET ligand is observed in this molecular docking analysis.

Additionally, the distances from the nucleophilic hydroxyl group of the respective catalytic serines to the closest 2PET carbonyl carbon ranged from 3.1 Å to 4.9 Å. The shortest, most catalytically viable distance was attributed to the Umep1:2PET conformer, whereas the longest distance was observed in the Umep2:2PET conformer (Supplementary Figures S4A–D).

## 4 Discussion

The identification of potential PET hydrolases in Antarctic rhizospheres provides new insights into the natural degradation pathways of synthetic polymers. PET is a persistent and widespread plastic pollutant that accumulates in soils and rhizospheres, serving as a potential xenobiotic carbon source for rhizospheric bacteria and influencing microbial enzymatic adaptation (Wang et al., 2024). By analyzing the rhizosphere soils of *Deschampsia antarctica* and *Colobanthus quitensis*, we focused on exploring these cold environments based on the evidence of reported hydrolytic enzyme activity against natural polyesters, such as cutin and suberin in temperate rhizospheres (Jadhav et al., 2017; Pellegrino et al., 2025; Saberi Riseh et al., 2024). We hypothesized that these enzymatic functions may also facilitate the degradation of synthetic polymers such as PET. This is supported by structural similarities between natural esters and PET’s aromatic ester bonds, as well as evidence that some PET hydrolases exhibit substrate promiscuity, acting on various ester substrates including *δ*-lactones and *β*-lactams (Zhang et al., 2024).

Metagenomic analysis employing a curated Hidden Markov Model identified 152 candidate hydrolases across 19 protein families, including established PET-active groups such as cutinases and carboxylesterases (Figure 2). The broad distribution of these enzymes in soil and rhizosphere environments demonstrates the potential of these habitats to degrade polyester substrates (Figure 1). Most candidates exhibited limited sequence similarity to previously characterized PET hydrolases used to develop the HMM (Erickson et al., 2022; Sulaiman et al., 2012; Yoshida et al., 2016) (Figure 3), suggesting that Antarctic rhizospheres may harbor novel enzyme structures with uncharacterized catalytic properties. This sequence diversity is environmentally important because enzymes with different structures may differ in substrate accessibility, stability, or efficiency under the cold, oligotrophic conditions typical of Antarctic soils. This variation affects PET degradation in the environment and offers new insights into the enzymatic potential of soil microbial communities in extreme environments.

Because many metabolic enzymes possess an *α*/β hydrolase fold or Ser-His-Asp triad without necessarily exhibiting PET hydrolase activity (Holmquist, 2000; Urvoy et al., 2021), we also focused on structural motifs. This approach identified four pPETHs containing the dienelactone hydrolase (DLH) fold and the canonical Ser-His-Asp catalytic triad, both of which are indicative of PET depolymerization activity (Alam et al., 2020). Our structural and docking analyses reveal that while the identified Antarctic pPETHs exhibit significant structural divergence from mesophilic models, most notably Acp1, they retain localized mechanisms crucial for substrate stabilization. For instance, Dup1 possesses homologous tryptophan residues (Trp123 and Trp149) known to facilitate aromatic interactions in *Is*PETase (Burgin et al., 2024; Chen et al., 2018). This stabilization in *Is*PETase is known to be carried out by *π*-π stacking interactions between the aromatic rings of Trp 159 Trp185 of *Is*PETase 6EQE and one of the aromatic rings of polyethylene terephthalate. However, our docking models did not capture the expected π − π stacked conformation between Trp123 or Trp149 to the 2PET ligand for this enzyme (Supplementary Table S7). This discrepancy is likely an artifact of utilizing the apo-state MD centroid, as PETases frequently undergo significant induced-fit conformational shifts upon substrate binding that static docking cannot fully capture (Austin et al., 2018; Guo et al., 2022).

Conversely, the favorable energies inferred from the docking analysis of the 2PET dimer into Acp1 highlights a remarkable evolutionary flexibility in catalytic architecture. Despite lacking the canonical aromatic stabilization and disulfide bonds, which at least one is expected for type I PETases (Joo et al., 2018), and exhibiting massive global structural deviation from *Is*PETase, Acp1 achieves a highly favorable nucleophilic attack geometry. The Ser-carbonyl distance of 3.7 Å is notably shorter than the typical distances reported for wild-type *Is*PETase and other homologous hydrolases, which generally range around 3.8–5.1 Å (Austin et al., 2018; Fecker et al., 2018). This tight catalytic distance, coupled with putative oxyanion hole stabilization (Ser34 and Leu112), suggests that despite the Acp1 structural divergence to *Is*PETase, local active-site geometry and interaction network may stabilize PET. The absence of the stabilizing disulfide bonds, which are key for structural stability in type I/II PETases like *Is*PETase and its homologs (Joo et al., 2018; Yoshida et al., 2016), signals that Acp1 and Dup1 do not fall into these categories. This structural divergence is supported by previous findings, such as the GuaPA archaeal PETase, which depolymerizes PET despite lacking a disulfide bond and is instead predicted to be stabilized by an extensive network of aromatic (π–π) interactions (Acosta et al., 2025). Recent findings further demonstrate that PET catalysis can occur in enzymes lacking disulfide bonds (Acosta et al., 2025; Mabashi-Asazuma et al., 2025). This reliance on non-covalent networks for stability is, furthermore, an expected characteristic of cold-adapted proteins. Psychrophilic enzymes typically rely on local dynamic non-covalent interactions to provide sufficient structural integrity without compromising the conformational flexibility required for efficient catalysis at low temperatures (Kulathunga and Potoyan, 2025).

Furthermore, the distinct structural dynamics of these enzymes may underscore a classic psychrophilic adaptation strategy: the trade-off between localized catalytic flexibility and global stability (Åqvist, 2017; Isaksen et al., 2014; Siddiqui and Cavicchioli, 2006) (Supplementary Figure S3). The localized relatively higher flexibility flanking the catalytic triad in Acp1 mimics the cold-adapted signatures seen in other environmental enzymes (Kulathunga and Potoyan, 2025; Siddiqui and Cavicchioli, 2006), potentially lowering the activation energy barrier for PET hydrolysis at low temperatures. In contrast, the global structural instability of Umep1 observed at 300 K reflects the inherent thermal fragility of naturally cold-adapted proteins when exposed to mesophilic conditions. Ultimately, while these *in silico* dynamics reveal compelling patterns of functional conservation and cold adaptation within Antarctic rhizosphere communities, the actual catalytic efficiency of these enzymes for PET depolymerization strictly requires empirical validation through *in vitro* biochemical assays.

Understanding the ecological and evolutionary context of these enzymes is critical for elucidating their environmental roles. Bacterial DLH enzymes are commonly involved in xenobiotic degradation pathways, particularly those processing chlorocatechols, where they hydrolyze dienelactones to produce maleylacetate (Maltseva et al., 1994; Moiseeva et al., 2002). The discovery of DLH-like hydrolases with a Ser-His-Asp triad, as opposed to the ancestral reconstructed Cys-His-Asp configuration (Song et al., 2025), suggests possible adaptation driven by selective pressures from synthetic polyesters. This hypothesis is supported by an ancestral sequence reconstruction study indicating that early PET-degrading enzymes may have originated from cysteine-based hydrolases (Song et al., 2025). In Antarctic rhizospheres, where microplastics and related chemical pollutants (Aves et al., 2022; Egas et al., 2025) may accumulate, such adaptations could represent microbial responses to emerging contaminants.

These findings underscore three principal ecological and biotechnological implications for soil enzyme diversity and function. First, Antarctic rhizospheres may serve as reservoirs of enzymatic diversity capable of degrading PET and related polymers, indicating a natural mechanism for reducing plastic pollutants in cold environments. Second, the novelty of the identified hydrolases demonstrates that significant portions of the enzymatic landscape relevant to pollutant degradation remain uncharacterized, including in plant-associated soils. Third, detailed knowledge of the catalytic properties and environmental distribution of these enzymes is crucial for predicting the persistence of PET in soils and assessing its interactions with other contaminants at the soil–plant interface.

In summary, although the primary function of these rhizosphere hydrolases is likely the breakdown of natural plant-derived polyesters, their structural characteristics indicate a latent capacity to degrade synthetic polymers such as PET. From both ecological and biotechnological perspectives, the diversity of these hydrolases advances the understanding of the soil enzymatic repertoire and its ability to metabolize non-native carbon sources. The detection of these sequences in Antarctic rhizospheres suggests that cold-soil microbiomes possess inherent capabilities for xenobiotic transformation, underscoring the importance of soil enzyme diversity for natural resilience and for the development of sustainable biotechnological applications. Future research integrating biochemical validation with soil activity assessments will clarify the role of these enzymes in carbon cycling and their potential as cold-active biocatalysts.

## 5 Conclusion

Soils from Livingston Island, particularly the rhizospheres of *Deschampsia antarctica* and *Colobanthus quitensis*, harbor microbial communities encoding PET-hydrolase-like enzymes that retain the conserved catalytic architecture of known polyester hydrolases. These findings unveil a previously overlooked dimension of enzymatic potential in cold-environment soils, underscoring the capacity of rhizosphere microbiomes to encode functionally diverse enzymes potentially active on novel carbon substrates. The identification of these genes and their predicted enzymes advances the understanding of soil microbial functional diversity and presents new opportunities for soil biotechnology and the development of cold-active enzymes for plastic bioremediation.

## Data Availability

The datasets presented in this study can be found in online repositories. The names of the repository/repositories and accession number(s) can be found in the article/Supplementary material.
