# Supplementary material for: Metagenomic insights into potential PET hydrolases from Antarctic soils and rhizospheres
Source: Front Microbiol. 2026 Jul 16;17:1749101. doi: 10.3389/fmicb.2026.1749101 (PMC13422221; doi:10.3389/fmicb.2026.1749101)
Supplement: Supplementary file 1 [file Data_Sheet_1.PDF]

Supplementary material

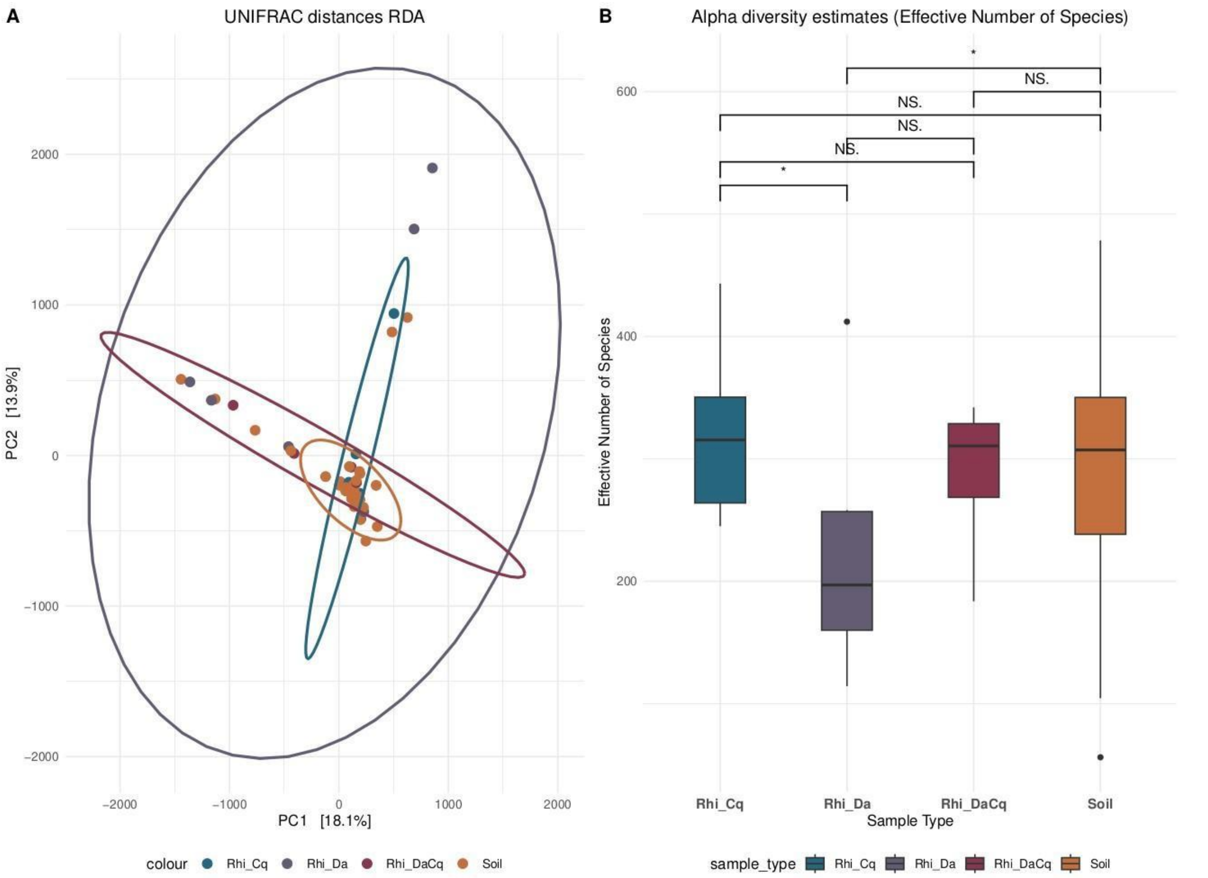

Supplementary Figure S1: Biodiversity measures across all collected sample types. A) Redundancy analysis (RDA) ordination plot of UNIFRAC distances between all 49 bacterial communities. From the MAGs-derived communities composition matrix, ecological distances weighted by phylogenetic distances were computed. The dispersion of samples in the biplot shows no apparent clustering between groups of the sample types, nevertheless, soil bacterial communities tend to cluster together; On the other hand, PERMANOVA analysis revealed that the sample types (rhizospheres of vascular plants and bulk soil) were a significant factor driving beta diversity between bacterial communities. **B)** The Shannon-Wiener index was calculated alongside the beta diversity analysis. The Effective Number of Species (ENS) was derived from these diversity indices. Pairwise Wilcoxon tests were performed on the ENS values from the different sample types. Significant differences (p-value < 0.05) were found only between two pairs: the rhizosphere bacterial communities of *Colobanthus quitensis* (Rhi_Cq) and *Deschampsia antarctica* (Rhi_Da), and between the bulk soil (Soils) and the *Deschampsia antarctica* rhizosphere (Rhi_Da).

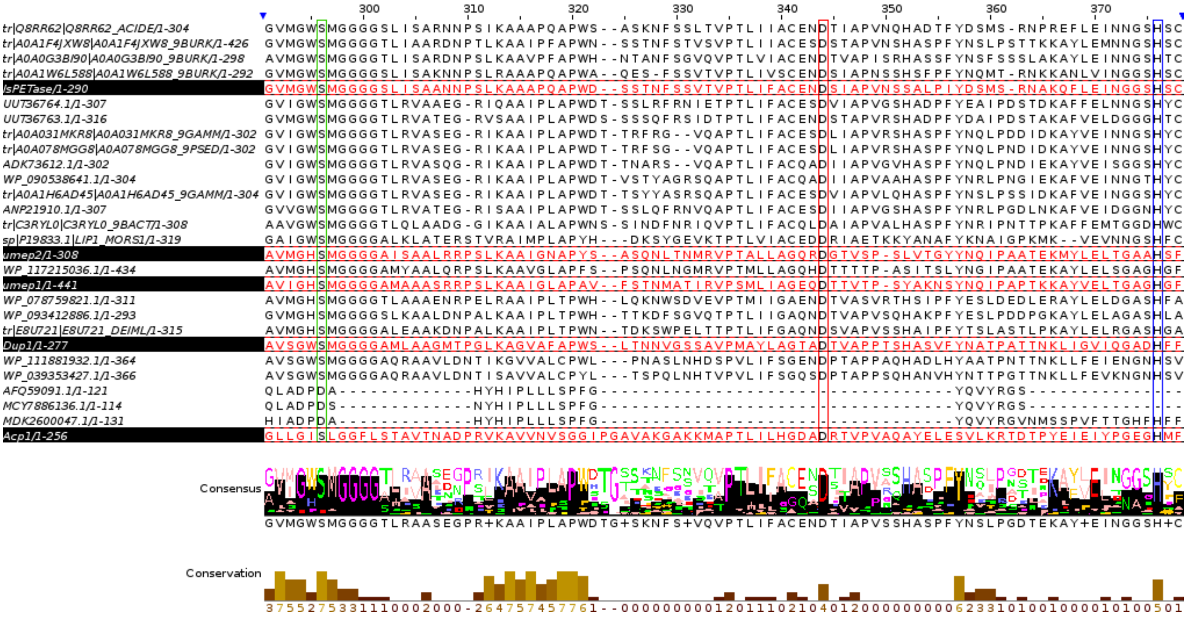

Supplementary Figure S2: Multiple sequence alignment of DLH-containing enzymes. Clustal Omega multiple sequence alignment of DLH domain-carrying enzymes detected by means of the HMM. Rows highlighted in black represent the identified candidate PET hydrolases from the Antarctic soils and rhizospheres of Deschampsia antarctica and *Colobanthus quitensis*. *Is*PETase is also highlighted in black. The other sequences correspond to the PAZy database PET enzymes with the detected DLH domain, as identified by the Pfam database. Columns hallmarked in green, red, and blue correspond to the Ser-Asp-His residues responsible for PET depolymerization catalysis in the *Is*PETase enzyme.

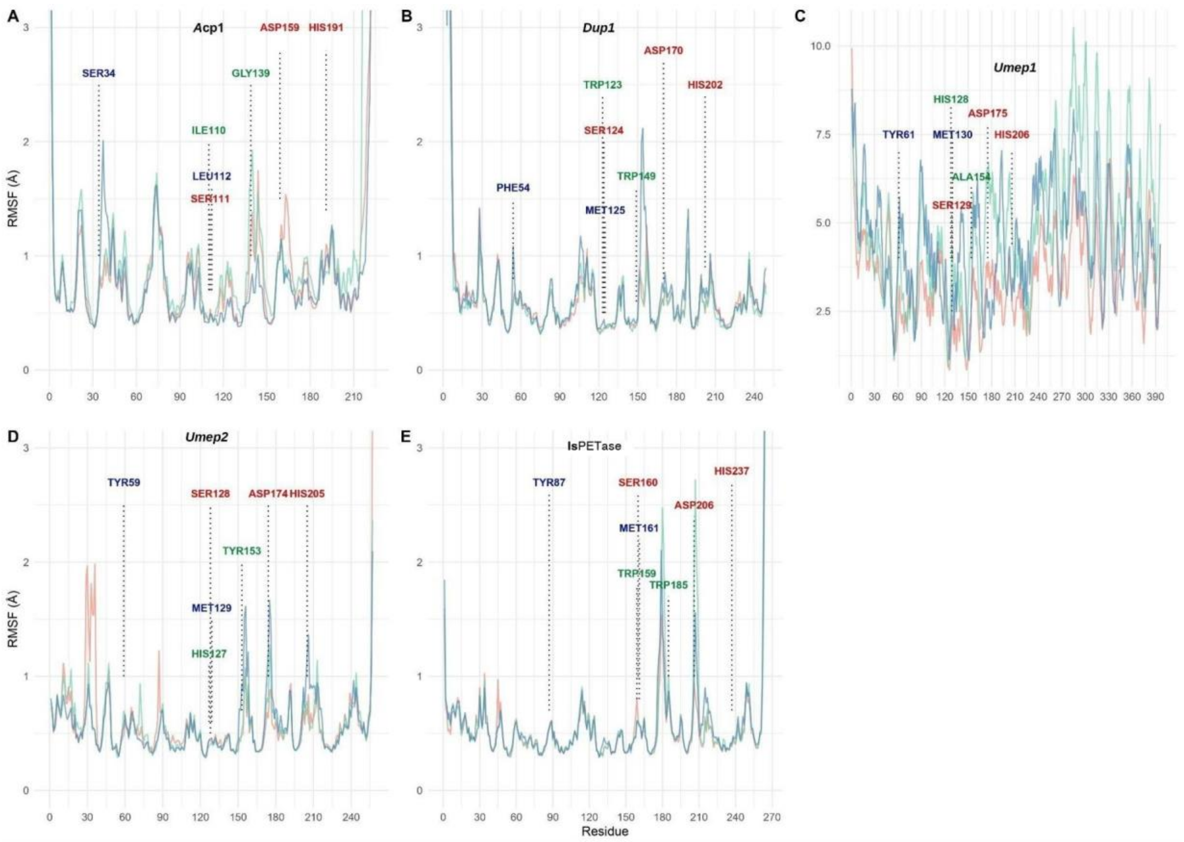

Supplementary Figure S3: Root mean square fluctuations (RMSF) during 100 ns molecular dynamics simulations. RMSF values were measured in triplicate for four putative PET hydrolases; (A) Acp1, (B) Dup1, (C) Umep1, and (D) Umep2; as well as (E) the reference *Is*PETase. Independent replicates 1, 2, and 3 are represented by red, green, and blue lines, respectively. The y-axis displays the RMSF in angstroms (Å) with a maximum scale of 3 Å for all structures, except for Umep1. Crucial residues are highlighted across all structures: the Ser-His-Asp catalytic triad is labeled in red; homologs of the *Is*PETase stabilizing tryptophan binding-site residues are highlighted in green; and homologs of the *Is*PETase oxyanion hole residues are highlighted in blue.

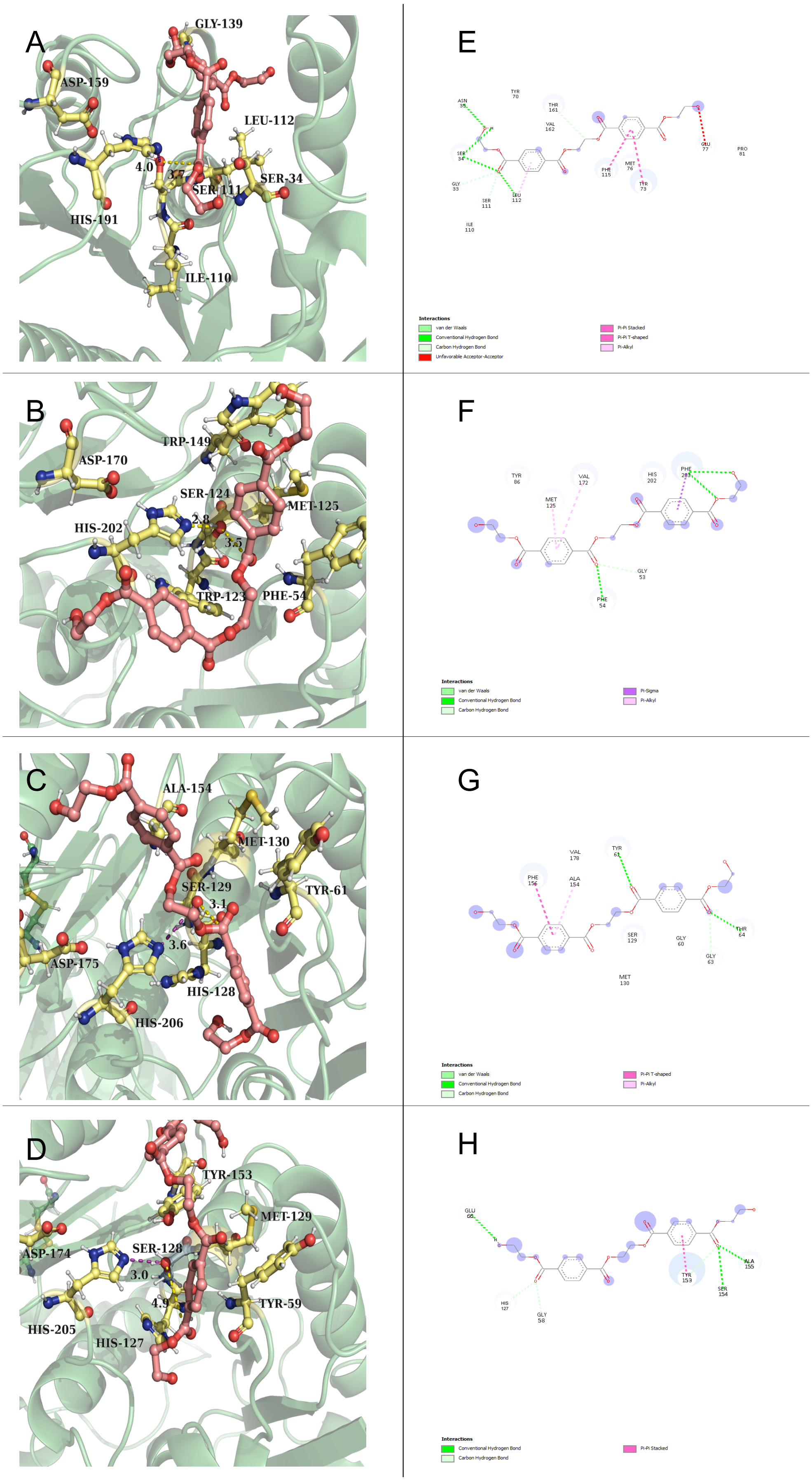
A
GLY-139
ASP-159
LEU-112
4.0
3.7
SER-111
SER-34
HIS-191
ILE-110
B
TRP-149
ASP-170
SER-124
MET-125
HIS-202
2.8
3.5
TRP-123
PHE-54
C
ALA-154
MET-130
SER-129
TYR-61
3.1
3.6
ASP-175
HIS-128
HIS-206
D
TYR-153
MET-129
SER-128
ASP-174
3.0
4.9
TYR-59
HIS-205
HIS-127
E
F
G
H
Interactions
van der Waals
Conventional Hydrogen Bond
Carbon Hydrogen Bond
Unfavorable Acceptor-Acceptor
Pi-Pi Stacked
Pi-Pi T-shaped
Pi-Alkyl
Pi-Sigma

Supplementary Figure S4: Molecular docking of putative PET hydrolases with a 2PET dimer. From A-D, protein-ligand complexes of Acp1, Dup1,Umep1 and Umep2 protein structures with 2PET (ethylene glycol terephthalate (3:2)). The protein structure frames corresponding to the centroid of the most populated cluster of the respective molecular dynamics were used as protein conformers for the molecular docking analysis. The 2PET dimer ligand is appreciated in salmon color. Key residues, including the catalytic Ser-His-Asp catalytic triad, oxyanion residues and ligand-stabilizing residues are highlighted in yellow color. Also, the distance between one of the PET carbonyl carbons and the oxygen of the catalytic serine is displayed in purple color. Finally, the distance of the histidine epsilon nitrogen is displayed in cyan color for all protein structures (A-D). From E-H, 2D interactio n maps are displayed for the respective four candidate proteins. Interactions distances from protein residues to the 2PET ligand are inferred from standard settings of BIOVIA Discovery Studio software.

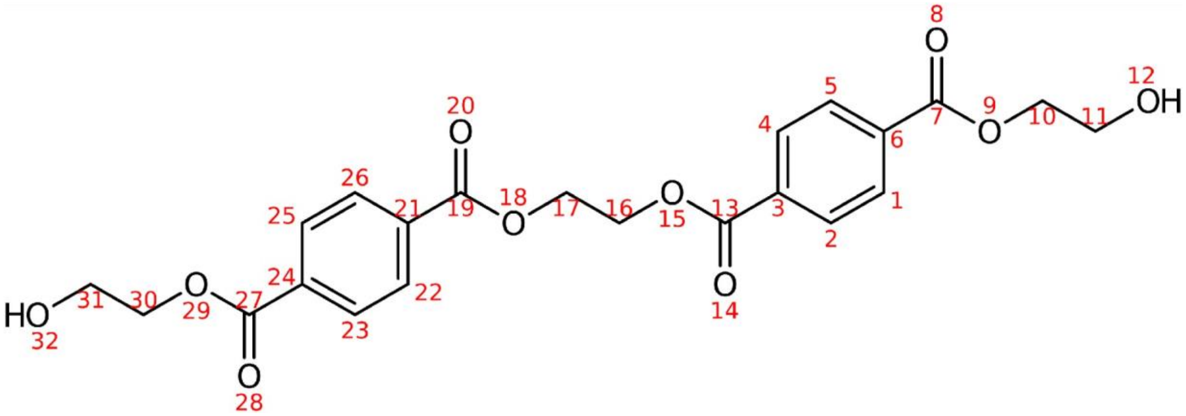

Supplementary Figure S5: 2PET (ethylene glycol terephthalate (3:2)) chemical structure used for docking analysis.

Supplementary Table S1: Samples metadata and sequencing data: Geolocation of samples retrieved from Byers Peninsula (62°S) is displayed along with DNA concentrations and the number or reads produced in Illumina NovaSeq 6000 platform. Reads retrieved after quality filtering are also shown.

**Sample**	Sample name	Number of raw sequences	Number of filtered sequences	Collection date	Location	Layout	Number of cycles	DNA concentration (ng/µl)	DNA Volume (µL)
SAMN37813271	ASB1	73097182	55034050	2019-02	62.670986 S 61.17595 W	paired-end	150	200	100
SAMN37813280	ASB10	73324114	56406182	2019-02	62.670993 S 61.175957 W	paired-end	150	52	100
SAMN37813281	ASB11	55686582	43384774	2019-02	62.670992 S 61.175956 W	paired-end	150	19	100
SAMN37813282	ASB12	84056840	64448308	2019-02	62.670996 S 61.17596 W	paired-end	150	200	100
SAMN37813283	ASB13	78266536	59704228	2019-02	62.670995 S 61.175959 W	paired-end	150	51	100
SAMN37813284	ASB14	74215676	59093124	2019-02	62.670997 S 61.175961 W	paired-end	150	29	100
SAMN37813285	ASB15	52293112	38665998	2019-02	62.671 S 61.175964 W	paired-end	150	20	100
SAMN37813286	ASB16	78197650	60323546	2019-02	62.670999 S 61.175963 W	paired-end	150	200	100
SAMN37813287	ASB17	77007406	59995200	2019-02	62.670998 S 61.175962 W	paired-end	150	51	100
SAMN37813288	ASB18	69619510	52277370	2019-02	62.61816635 S 61.04946346 W	paired-end	150	40	100
SAMN37813289	ASB19	57248776	44727468	2019-02	62.61816634 S 61.04946345 W	paired-end	150	22	100
SAMN37813272	ASB2	65835522	49502618	2019-02	62.670985 S 61.175949 W	paired-end	150	56	100
SAMN37813290	ASB20	75327146	56145938	2019-02	62.61816637 S 61.04946348 W	paired-end	150	53	100
SAMN37813291	ASB21	54504982	40722788	2019-02	62.61816636 S 61.04946347 W	paired-end	150	51	100
SAMN37813292	ASB22	90397502	70664378	2019-02	62.61816633 S 61.04946344 W	paired-end	150	14	100
SAMN37813293	ASB23	65301280	52524458	2019-02	62.6181664 S 61.04946351 W	paired-end	150	200	100
SAMN37813294	ASB24	76862330	58163202	2019-02	62.61816639 S 61.0494635 W	paired-end	150	200	100
SAMN37813295	ASB25	65274060	49910584	2019-02	62.61816638 S 61.04946349 W	paired-end	150	26	100
SAMN37813296	ASB26	46524830	36368250	2019-02	62.672382 S 60.910434 W	paired-end	150	200	100
SAMN37813297	ASB27	68822544	53746132	2019-02	62.672381 S 60.910433 W	paired-end	150	30	100
SAMN37813298	ASB28	45057640	34965440	2019-02	62.672383 S 60.910435 W	paired-end	150	2	25
SAMN37813299	ASB29	76501924	58526070	2019-02	62.672386 S 60.910438 W	paired-end	150	57	100
SAMN37813273	ASB3	53727668	43781734	2019-02	62.670984 S 61.175948 W	paired-end	150	17	25
SAMN37813300	ASB30	62523118	49677716	2019-02	62.672385 S 60.910437 W	paired-end	150	26	100
SAMN37813301	ASB31	51583114	41766052	2019-02	62.672384 S 60.910436 W	paired-end	150	8	25
SAMN37813302	ASB32	77468862	58683510	2019-02	62.672389 S 60.910441 W	paired-end	150	36	100
SAMN37813303	ASB33	59551216	45890664	2019-02	62.672388 S 60.91044 W	paired-end	150	33	100
SAMN37813304	ASB34	36542230	30122768	2019-02	62.672387 S 60.910439 W	paired-end	150	16	25
SAMN37813305	ASB35	80700716	59775270	2019-02	62.6774367 S 60.895524 W	paired-end	150	200	100
SAMN37813306	ASB36	67559364	51449534	2019-02	62.6774366 S 60.895523 W	paired-end	150	16	100
SAMN37813307	ASB37	64720724	49547974	2019-02	62.6774365 S 60.895522 W	paired-end	150	2	25
SAMN37813308	ASB38	60611884	46090120	2019-02	62.677437 S 60.895527 W	paired-end	150	200	100
SAMN37813309	ASB39	76255066	59308340	2019-02	62.6774369 S 60.895526 W	paired-end	150	29	100
SAMN37813274	ASB4	92012394	69026906	2019-02	62.670989 S 61.175953 W	paired-end	150	200	100
SAMN37813310	ASB40	74102772	57842920	2019-02	62.6774368 S 60.895525 W	paired-end	150	3	25
SAMN37813311	ASB41	78414092	59217306	2019-02	62.6774373 S 60.89553 W	paired-end	150	59	100
SAMN37813312	ASB42	68796514	52365272	2019-02	62.6774372 S 60.895529 W	paired-end	150	23	100
SAMN37813313	ASB43	65473930	50025442	2019-02	62.6774371 S 60.895528 W	paired-end	150	11	100
SAMN37813314	ASB44	69891250	29947496	2019-02	62.6774375 S 60.895532 W	paired-end	150	29	100
SAMN37813315	ASB45	59383930	46653990	2019-02	62.6774374 S 60.895531 W	paired-end	150	21	100
SAMN37813316	ASB46	41984126	34664306	2019-02	62.6774376 S 60.895533 W	paired-end	150	4	25
SAMN37813317	ASB47	85228376	27044608	2019-02	62.6774379 S 60.895536 W	paired-end	150	200	100
SAMN37813318	ASB48	69087794	52104392	2019-02	62.6774378 S 60.895535 W	paired-end	150	200	100
SAMN37813319	ASB49	68940098	53778302	2019-02	62.6774377 S 60.895534 W	paired-end	150	55	100

SAMN37813275	ASB5	62207084	48403364	2019-02	62.670988 S 61.175952 W	paired-end	150	25	100
SAMN37813276	ASB6	70195456	54323748	2019-02	62.670987 S 61.175951 W	paired-end	150	27	100
SAMN37813277	ASB7	92219820	70603524	2019-02	62.670991 S 61.175955 W	paired-end	150	200	100
SAMN37813278	ASB8	83709788	64123324	2019-02	62.67099 S 61.175954 W	paired-end	150	200	100
SAMN37813279	ASB9	88258562	67262450	2019-02	62.670994 S 61.175958 W	paired-end	150	200	100

Supplementary Table S2: Pfam annotations associated with each one of the 152 detected putative PET hydrolases. Genome bacterial Phylum associated with the enzyme's bacterial host is also displayed. Proteins identified with the Dienelactone hydrolase family are highlighted in the "Candidate" column.

ProteinID	Annotation	Phylum	Candidate
prot1	Prolyl oligopeptidase family	Acidobacteriota	No
prot2	Serine aminopeptidase, S33	Gemmatimonadota	No
prot3	Serine aminopeptidase, S33	Actinobacteriota	No
prot4	BD-FAE	Verrucomicrobiota	No
prot5	BD-FAE	Gemmatimonadota	No
prot6	Prolyl oligopeptidase family	Actinobacteriota	No
prot7	Platelet-activating factor acetylhydrolase, isoform II	Bacteroidota	No
prot8	Platelet-activating factor acetylhydrolase, isoform II	Bacteroidota	No
prot9	Prolyl oligopeptidase family	Actinobacteriota	No
prot10	Serine aminopeptidase, S33	Myxococcota	No
prot11	BD-FAE	Bacteroidota	No
prot12	BD-FAE	Verrucomicrobiota	No
prot13	BD-FAE	Acidobacteriota	No
prot14	Prolyl oligopeptidase family	Proteobacteria	No
prot15	BD-FAE	Gemmatimonadota	No
prot16	BD-FAE	Gemmatimonadota	No
prot17	Serine aminopeptidase, S33	Actinobacteriota	No
prot18	Platelet-activating factor acetylhydrolase, isoform II	Acidobacteriota	No
prot19	BD-FAE	Acidobacteriota	No
prot20	Platelet-activating factor acetylhydrolase, isoform II	Bacteroidota	No

prot21	Platelet-activating factor acetylhydrolase, isoform II	Bacteroidota	No
prot22	Platelet-activating factor acetylhydrolase, isoform II	Bacteroidota	No
prot23	Prolyl oligopeptidase family	Gemmatimonadota	No
prot24	Prolyl oligopeptidase family	Acidobacteriota	No
prot25	Serine aminopeptidase, S33	Gemmatimonadota	No
prot26	BD-FAE	Acidobacteriota	No
prot27	Cutinase	Bacteroidota	No
prot28	BD-FAE	Myxococcota	No
prot29	BD-FAE	Gemmatimonadota	No
prot30	Cutinase	Chloroflexota	No
prot31	Platelet-activating factor acetylhydrolase, isoform II	Chloroflexota	No
prot32	BD-FAE	Proteobacteria	No
prot33	Serine aminopeptidase, S33	Chloroflexota	No
prot34	Platelet-activating factor acetylhydrolase, isoform II	Bacteroidota	No
prot35	Platelet-activating factor acetylhydrolase, isoform II	Gemmatimonadota	No
prot36	Prolyl oligopeptidase family	Acidobacteriota	No
prot37	BD-FAE	Gemmatimonadota	No
prot38	BD-FAE	Acidobacteriota	No
prot39	PQQ-like domain	Acidobacteriota	No
prot40	Cutinase	Desulfobacterota_B	No
prot41	BD-FAE	Bacteroidota	No
prot42	Serine aminopeptidase, S33	Myxococcota	No
prot43	Platelet-activating factor acetylhydrolase, isoform II	Acidobacteriota	No
prot44	WD40-like Beta Propeller Repeat	Acidobacteriota	No
prot45	Prolyl oligopeptidase family	Acidobacteriota	No
prot46	Secretion system C-terminal sorting domain	Bacteroidota	No
prot47	Cutinase	Bacteroidota	No
prot48	Secretion system C-terminal sorting domain	Bacteroidota	No
prot49	BD-FAE	Bacteroidota	No
Acp1	Dienelactone hydrolase family	Acidobacteriota	Yes
prot51	Serine aminopeptidase, S33	Actinobacteriota	No
prot52	Prolyl oligopeptidase family	Chloroflexota	No
prot53	Prolyl oligopeptidase family	Chloroflexota	No
prot54	Carboxylesterase family	Chloroflexota	No

prot55	Prolyl oligopeptidase family	Chloroflexota	No
prot56	WD40-like Beta Propeller Repeat	Chloroflexota	No
prot57	Carboxylesterase family	Chloroflexota	No
prot58	Prolyl oligopeptidase family	Chloroflexota	No
prot59	WD40-like Beta Propeller Repeat	Chloroflexota	No
prot60	Secretion system C-terminal sorting domain	Bacteroidota	No
prot61	Prolyl oligopeptidase family	Bacteroidota	No
prot62	Prolyl oligopeptidase family	Proteobacteria	No
prot63	Serine aminopeptidase, S33	Actinobacteriota	No
prot64	Prolyl oligopeptidase family	Chloroflexota	No
prot65	Cutinase	Actinobacteriota	No
prot66	Alpha/beta hydrolase of unknown function (DUF1400)	Cyanobacteria	No
prot67	Secretion system C-terminal sorting domain	Bacteroidota	No
prot68	Secretion system C-terminal sorting domain	Bacteroidota	No
prot69	Serine aminopeptidase, S33	Myxococcota	No
prot70	Alpha/beta hydrolase of unknown function (DUF1400)	Cyanobacteria	No
prot71	Serine aminopeptidase, S33	Myxococcota	No
prot72	Secretion system C-terminal sorting domain	Bacteroidota	No
prot73	Prolyl oligopeptidase family	Bacteroidota	No
prot74	ABC transporter	Actinobacteriota	No
prot75	X-Pro dipeptidyl-peptidase (S15 family)	Actinobacteriota	No
prot76	X-Pro dipeptidyl-peptidase C-terminal non-catalytic domain	Actinobacteriota	No
prot77	BD-FAE	Bacteroidota	No
prot78	Prolyl oligopeptidase family	Armatimonadota	No
prot79	Prolyl oligopeptidase family	Chloroflexota	No
prot80	WD40-like Beta Propeller Repeat	Chloroflexota	No
prot81	Carboxylesterase family	Chloroflexota	No
prot82	Prolyl oligopeptidase family	Chloroflexota	No
prot83	WD40-like Beta Propeller Repeat	Chloroflexota	No
prot84	Secretion system C-terminal sorting domain	Bacteroidota	No
prot85	Carboxylesterase family	Chloroflexota	No
prot86	Prolyl oligopeptidase family	Chloroflexota	No
prot87	Cutinase	Actinobacteriota	No

prot88	Secretion system C-terminal sorting domain	Bacteroidota	No
prot89	Prolyl oligopeptidase family	Proteobacteria	No
prot90	Prolyl oligopeptidase family	Chloroflexota	No
prot91	Serine aminopeptidase, S33	Verrucomicrobiota	No
prot92	alpha/beta hydrolase fold	Bacteroidota	No
prot93	Chlorophyllase	Myxococcota	No
prot94	Prolyl oligopeptidase family	Chloroflexota	No
prot95	Serine aminopeptidase, S33	Actinobacteriota	No
prot96	Cutinase	Myxococcota	No
prot97	Serine aminopeptidase, S33	Proteobacteria	No
prot98	Serine aminopeptidase, S33	Verrucomicrobiota	No
prot99	WD40-like Beta Propeller Repeat	Acidobacteriota	No
prot100	Prolyl oligopeptidase family	Acidobacteriota	No
prot101	Secretion system C-terminal sorting domain	Bacteroidota	No
prot102	Chlorophyllase	Actinobacteriota	No
prot103	Platelet-activating factor acetylhydrolase, isoform II	Proteobacteria	No
prot104	Serine aminopeptidase, S33	Actinobacteriota	No
prot105	BD-FAE	Proteobacteria	No
prot106	BD-FAE	Bacteroidota	No
prot107	Serine aminopeptidase, S33	Proteobacteria	No
prot108	Phospholipase/Carboxylesterase	Bacteroidota	No
prot109	BD-FAE	Bacteroidota	No
prot110	BD-FAE	Bacteroidota	No
prot111	Platelet-activating factor acetylhydrolase, isoform II	Bacteroidota	No
prot112	Platelet-activating factor acetylhydrolase, isoform II	Bacteroidota	No
prot113	BD-FAE	Bacteroidota	No
prot114	BD-FAE	Bacteroidota	No
prot115	Platelet-activating factor acetylhydrolase, isoform II	Bacteroidota	No
prot116	BD-FAE	Gemmatimonadota	No
prot117	Carboxylesterase family	Actinobacteriota	No
prot118	Prolyl oligopeptidase family	Proteobacteria	No
prot119	Platelet-activating factor acetylhydrolase, isoform II	Proteobacteria	No
prot120	Platelet-activating factor acetylhydrolase, isoform II	Bacteroidota	No
prot121	BD-FAE	Gemmatimonadota	No

prot122	Serine aminopeptidase, S33	Chloroflexota	No
Umep1	Dienelactone hydrolase family	Actinobacteriota	Yes
Umep2	Dienelactone hydrolase family	Actinobacteriota	Yes
prot125	X-Pro dipeptidyl-peptidase (S15 family)	Actinobacteriota	No
prot126	ABC transporter	Actinobacteriota	No
prot127	X-Pro dipeptidyl-peptidase C-terminal non-catalytic domain	Actinobacteriota	No
prot128	BD-FAE	Bacteroidota	No
prot129	BD-FAE	Bacteroidota	No
prot130	BD-FAE	Bacteroidota	No
prot131	alpha/beta hydrolase fold	Bacteroidota	No
prot132	WD40-like Beta Propeller Repeat	Acidobacteriota	No
prot133	Prolyl oligopeptidase family	Acidobacteriota	No
prot134	Putative esterase	Proteobacteria	No
prot135	Carboxylesterase family	Proteobacteria	No
prot136	Prolyl oligopeptidase family	Proteobacteria	No
prot137	BD-FAE	Bacteroidota	No
prot138	BD-FAE	Bacteroidota	No
prot139	Serine aminopeptidase, S33	Proteobacteria	No
prot140	Putative esterase	Proteobacteria	No
prot141	Carboxylesterase family	Proteobacteria	No
prot142	Carboxylesterase family	Actinobacteriota	No
prot143	Prolyl oligopeptidase family	Chloroflexota	No
prot144	Alpha/beta hydrolase family	Acidobacteriota	No
prot145	Serine aminopeptidase, S33	Verrucomicrobiota	No
prot146	X-Pro dipeptidyl-peptidase (S15 family)	Actinobacteriota	No
prot147	ABC transporter	Actinobacteriota	No
prot148	X-Pro dipeptidyl-peptidase C-terminal non-catalytic domain	Actinobacteriota	No
prot149	Cutinase	Bacteroidota	No
prot150	Secretion system C-terminal sorting domain	Bacteroidota	No
prot151	Serine aminopeptidase, S33	Actinobacteriota	No
Dup1	Dienelactone hydrolase family	Proteobacteria	Yes

Supplementary Table S3: Supplementary Table S3 lists all nodes included in the Sequence Similarity Network (Figure 3) , both the 86 experimentally validated PAZy reference sequences and the putative PET hydrolases identified in this study ,and maps each sequence's original Pfam annotation (column 'Pfam annotation') to the simplified category used for network coloring (column 'Agglomerated annotation'). It therefore differs in scope and purpose from Supplementary Table S2, which catalogs only the 152 study-derived sequences together with their host phylum and candidate status.

gene ID	pfam annotation	Agglomerated annotation
tr|A0A031MKR8|A0A031MKR8_9GAMM	PET hydrolase-like	PET hydrolase-like
tr|A0A075B5G4|A0A075B5G4_HUMIN	Cutinase	Cutinase
tr|A0A078MGG8|A0A078MGG8_9PSED	PET hydrolase-like	PET hydrolase-like
WP_034767800.1	Serine aminopeptidase, S33	Serine aminopeptidase, S33
CEE00769.1	alpha/beta hydrolase fold	Alpha/beta hydrolase family
WP_039353427.1	Secretion system C-terminal sorting domain	Others
WP_041846030.1	Prolyl oligopeptidase family	Prolyl oligopeptidase family
WP_041847557.1	Carboxylesterase family	Carboxylesterase family
tr|A0A0G3BI90|A0A0G3BI90_9BURK	PET hydrolase-like	PET hydrolase-like
sp|A0A0K8P6T7|PETH_IDESA	PET hydrolase-like	PET hydrolase-like
ANP21910.1	PET hydrolase-like	PET hydrolase-like
tr|A0A1F4JXW8|A0A1F4JXW8_9BURK	PET hydrolase-like	PET hydrolase-like
WP_090538641.1	PET hydrolase-like	PET hydrolase-like
WP_093412886.1	PET hydrolase-like	PET hydrolase-like
SHM40309.1	PET hydrolase-like	PET hydrolase-like
WP_085749238.1	PET hydrolase-like	PET hydrolase-like
tr|A0A1W6L588|A0A1W6L588_9BURK	PET hydrolase-like	PET hydrolase-like
tr|A0A1Z2SIQ1|A0A1Z2SIQ1_VIBGA	PET hydrolase-like	PET hydrolase-like
GBD22443.1	PET hydrolase-like	PET hydrolase-like
WP_108898452.1	Lipase-like, C-terminal domain	Others
WP_111881932.1	Secretion system C-terminal sorting domain	Others
RLT92980.1	PET hydrolase-like	PET hydrolase-like
RLU00646.1	PET hydrolase-like	PET hydrolase-like
RLI42440.1	Prolyl oligopeptidase family	Prolyl oligopeptidase family
QEX94755.1	Cutinase	Cutinase
DAC80635.1	PET hydrolase-like	PET hydrolase-like
tr|C3RYL0|C3RYL0_9BACT	PET hydrolase-like	PET hydrolase-like

ACY95991.1	PET hydrolase-like	PET hydrolase-like
tr|D1A9G5|D1A9G5_THECD	PET hydrolase-like	PET hydrolase-like
BAI99230.2	PET hydrolase-like	PET hydrolase-like
ADH43200.1	Carboxylesterase family	Carboxylesterase family
tr|E5BBQ2|E5BBQ2_THEFU	PET hydrolase-like	PET hydrolase-like
tr|E8U721|E8U721_DEIML	PET hydrolase-like	PET hydrolase-like
ADK73612.1	PET hydrolase-like	PET hydrolase-like
tr|E9LVH7|E9LVH7_THEAE	PET hydrolase-like	PET hydrolase-like
ADV92526.1	PET hydrolase-like	PET hydrolase-like
ADV92527.1	PET hydrolase-like	PET hydrolase-like
EGD44994.1	PET hydrolase-like	PET hydrolase-like
sp|F7IX06|PETH2_THEAE	PET hydrolase-like	PET hydrolase-like
sp|G9BY57|PETH_UNKP	PET hydrolase-like	PET hydrolase-like
tr|H6WX58|H6WX58_9ACTN	PET hydrolase-like	PET hydrolase-like
AAA33335.1	Cutinase	Cutinase
sp|P19833|LIP1_MORS1	PET hydrolase-like	PET hydrolase-like
sp|P41365|LIPB_PSEA2	NA	Others
sp|Q47RJ7|PETH1_THEFY	PET hydrolase-like	PET hydrolase-like
ADM47605.1	PET hydrolase-like	PET hydrolase-like
tr|Q8RR62|Q8RR62_ACIDE	PET hydrolase-like	PET hydrolase-like
tr|R4YKL9|R4YKL9_OLEAN	PET hydrolase-like	PET hydrolase-like
ESK97883.1	Cutinase	Cutinase
tr|W0TJ64|W0TJ64_9PSEU	PET hydrolase-like	PET hydrolase-like
tr|W6R2Y2|W6R2Y2_PSEP5	GDSL-like Lipase/Acylhydrolase family	Others
tr|X0BTD8|X0BTD8_FUSOX	Cutinase	Cutinase
cluster1_bin.562_k127_13515535_5	Serine aminopeptidase, S33	Serine aminopeptidase, S33
cluster1_bin.555_k127_1912875_3	NA	Others
cluster1_bin.555_k127_15813091_5	NA	Others
cluster1_bin.55_k127_9501638_11	BD-FAE	BD-FAE
cluster1_bin.468_k127_9785410_113	BD-FAE	BD-FAE
cluster1_bin.445_k127_2483607_9	Serine aminopeptidase, S33	Serine aminopeptidase, S33
cluster1_bin.444_k127_13043240_4	Phospholipase/Carboxylesterase	Others
cluster1_bin.434_k127_11312256_4	BD-FAE	BD-FAE
cluster1_bin.433_k127_13468669_8	Platelet-activating factor acetylhydrolase, isoform II	Platelet-activating factor acetylhydrolase, isoform II

cluster1_bin.430_k127_13468669_8	Platelet-activating factor acetylhydrolase, isoform II	Platelet-activating factor acetylhydrolase, isoform II
cluster1_bin.427_k127_159818_9	BD-FAE	BD-FAE
cluster1_bin.427_k127_14917376_2	BD-FAE	BD-FAE
cluster1_bin.421_k127_13757179_52	Platelet-activating factor acetylhydrolase, isoform II	Platelet-activating factor acetylhydrolase, isoform II
cluster1_bin.406_k127_14640715_16	NA	Others
cluster1_bin.392_k127_7981816_13	BD-FAE	BD-FAE
cluster1_bin.389_k127_1565834_30	Carboxylesterase family	Carboxylesterase family
cluster1_bin.388_k127_5075700_3	Prolyl oligopeptidase family	Prolyl oligopeptidase family
cluster1_bin.385_k127_11671208_263	Platelet-activating factor acetylhydrolase, isoform II	Platelet-activating factor acetylhydrolase, isoform II
cluster1_bin.374_k127_13757179_52	Platelet-activating factor acetylhydrolase, isoform II	Platelet-activating factor acetylhydrolase, isoform II
cluster1_bin.366_k127_34855_10	NA	Others
cluster1_bin.362_k127_1912875_3	NA	Others
cluster1_bin.362_k127_15813091_5	NA	Others
cluster1_bin.359_k127_7981816_13	BD-FAE	BD-FAE
cluster1_bin.350_k127_14640715_16	NA	Others
cluster1_bin.349_k127_11021533_3	Dienelactone hydrolase family	Dienelactone hydrolase family
cluster1_bin.349_k127_11021533_3	NA	NA
cluster1_bin.333_k127_7292371_2	Serine aminopeptidase, S33	Serine aminopeptidase, S33
cluster1_bin.324_k127_8005854_2	NA	Others
cluster1_bin.324_k127_2448869_2	NA	Others
cluster1_bin.324_k127_13009386_2	Dienelactone hydrolase family	Dienelactone hydrolase family
cluster1_bin.324_k127_10909236_1	Dienelactone hydrolase family	Dienelactone hydrolase family
cluster1_bin.307_k127_13004062_6	X-Pro dipeptidyl-peptidase (S15 family)	Others
cluster1_bin.3_k127_11312256_4	BD-FAE	BD-FAE
cluster1_bin.277_k127_159818_9	BD-FAE	BD-FAE
cluster1_bin.277_k127_14917376_2	BD-FAE	BD-FAE
cluster1_bin.273_k127_9630764_289	alpha/beta hydrolase fold	Alpha/beta hydrolase family
cluster1_bin.263_k127_13795383_2	Putative esterase	Putative esterase
cluster1_bin.223_k127_5075700_3	Prolyl oligopeptidase family	Prolyl oligopeptidase family
cluster1_bin.219_k127_10398450_2	BD-FAE	BD-FAE
cluster1_bin.218_k127_10398450_2	BD-FAE	BD-FAE
cluster1_bin.217_k127_2483607_9	Serine aminopeptidase, S33	Serine aminopeptidase, S33
cluster1_bin.210_k127_13795383_2	Putative esterase	Putative esterase
cluster1_bin.208_k127_1565834_30	Carboxylesterase family	Carboxylesterase family

cluster1_bin.170_k127_2142616_5	Prolyl oligopeptidase family	Prolyl oligopeptidase family
cluster1_bin.162_k127_4512784_6	Alpha/beta hydrolase family	Alpha/beta hydrolase family
cluster1_bin.157_k127_4459843_4	Serine aminopeptidase, S33	Serine aminopeptidase, S33
cluster1_bin.150_k127_13004062_6	X-Pro dipeptidyl-peptidase (S15 family)	Others
cluster1_bin.139_k127_6368924_3	NA	Others
cluster1_bin.128_k127_6830642_6	Cutinase	Cutinase
cluster1_bin.108_k127_13515535_5	Serine aminopeptidase, S33	Serine aminopeptidase, S33
cluster1_bin.107_k127_8005854_2	NA	Others
cluster1_bin.107_k127_2448869_2	NA	Others
UUT36764.1	PET hydrolase-like	PET hydrolase-like
UUT36763.1	PET hydrolase-like	PET hydrolase-like
tr|A0A1H6AD45|A0A1H6AD45_9GAMM	PET hydrolase-like	PET hydrolase-like
pdb|2FX5|A	PET hydrolase-like	PET hydrolase-like
SUD16364.1	PET hydrolase-like	PET hydrolase-like
MGYP000532440779	PET hydrolase-like	PET hydrolase-like
WP_085690612.1	Phospholipase/Carboxylesterase	Others
WP_101893509.1	PET hydrolase-like	PET hydrolase-like
ODU60407.1	PET hydrolase-like	PET hydrolase-like
WP_062195544.1	PET hydrolase-like	PET hydrolase-like
WP_083947829.1	PET hydrolase-like	PET hydrolase-like
WP_082414832.1	PET hydrolase-like	PET hydrolase-like
WP_117215036.1	PET hydrolase-like	PET hydrolase-like
WP_078759821.1	PET hydrolase-like	PET hydrolase-like
WP_107095481.1	PET hydrolase-like	PET hydrolase-like
UNZ22463.1	Cutinase	Cutinase
SAY37579.1	PET hydrolase-like	PET hydrolase-like
SAY37582.1	PET hydrolase-like	PET hydrolase-like
WAU86704.1	PET hydrolase-like	PET hydrolase-like
WOR09923.1	PET hydrolase-like	PET hydrolase-like
pdb|7NEI|B	PET hydrolase-like	PET hydrolase-like
pdb|7CUV|A	PET hydrolase-like	PET hydrolase-like
MBO2503201.1	PET hydrolase-like	PET hydrolase-like
SAY37592.1	PET hydrolase-like	PET hydrolase-like
SAY37583.1	PET hydrolase-like	PET hydrolase-like

AKZ20828.1	Lipase-like, C-terminal domain	Others
WP_108898647.1	Serine aminopeptidase, S33	Serine aminopeptidase, S33
UNZ81746.1	alpha/beta hydrolase fold	Alpha/beta hydrolase family
UNZ81748.1	alpha/beta hydrolase fold	Alpha/beta hydrolase family
UNZ81747.1	Beta-lactamase	Others
SAY37584.1	PET hydrolase-like	PET hydrolase-like
SAY37587.1	PET hydrolase-like	PET hydrolase-like
SAY37589.1	PET hydrolase-like	PET hydrolase-like
cluster4_bin.201_NODE_6270_length_17733_cov_8.196968_9	NA	Others
cluster4_bin.197_NODE_7028_length_16706_cov_15.148460_12	Serine aminopeptidase, S33	Serine aminopeptidase, S33
cluster4_bin.196_NODE_39347_length_6692_cov_4.663854_4	BD-FAE	BD-FAE
cluster4_bin.187_NODE_15688_length_10962_cov_9.489410_9	BD-FAE	BD-FAE
cluster4_bin.17_NODE_36530_length_6963_cov_5.863781_3	BD-FAE	BD-FAE
cluster4_bin.164_NODE_32147_length_7456_cov_5.854614_5	Prolyl oligopeptidase family	Prolyl oligopeptidase family
cluster4_bin.139_NODE_85768_length_4446_cov_8.326349_4	BD-FAE	BD-FAE
cluster4_bin.128_NODE_34584_length_7167_cov_5.989314_4	BD-FAE	BD-FAE
cluster4_bin.126_NODE_1669_length_33111_cov_22.671981_33	Serine aminopeptidase, S33	Serine aminopeptidase, S33
cluster3_bin.82_NODE_51273_length_7337_cov_5.531859_3	Platelet-activating factor acetylhydrolase, isoform II	Platelet-activating factor acetylhydrolase, isoform II
cluster3_bin.71_NODE_13612_length_16296_cov_6.931408_9	BD-FAE	BD-FAE
cluster3_bin.63_NODE_4456_length_30491_cov_23.912899_11	Platelet-activating factor acetylhydrolase, isoform II	Platelet-activating factor acetylhydrolase, isoform II
cluster3_bin.56_NODE_57_length_203467_cov_77.721049_19	Platelet-activating factor acetylhydrolase, isoform II	Platelet-activating factor acetylhydrolase, isoform II
cluster3_bin.4_NODE_3_length_576445_cov_12.648606_375	Platelet-activating factor acetylhydrolase, isoform II	Platelet-activating factor acetylhydrolase, isoform II
cluster3_bin.34_NODE_59365_length_6712_cov_13.198588_3	Prolyl oligopeptidase family	Prolyl oligopeptidase family
cluster3_bin.294_NODE_19846_length_13082_cov_4.631918_8	NA	Others
cluster3_bin.294_NODE_194205_length_3243_cov_3.830301_1	WD40-like Beta Propeller Repeat	Others
cluster3_bin.29_NODE_3946_length_32540_cov_15.616192_25	Serine aminopeptidase, S33	Serine aminopeptidase, S33
cluster3_bin.282_NODE_74826_length_5825_cov_6.017158_3	BD-FAE	BD-FAE
cluster3_bin.257_NODE_95172_length_5023_cov_3.873390_4	Cutinase	Cutinase
cluster3_bin.226_NODE_117249_length_4419_cov_4.148946_4	BD-FAE	BD-FAE
cluster3_bin.21_NODE_42263_length_8252_cov_11.957057_3	BD-FAE	BD-FAE
cluster3_bin.208_NODE_81503_length_5523_cov_9.454645_3	Cutinase	Cutinase
cluster3_bin.208_NODE_10654_length_18757_cov_8.333708_6	Platelet-activating factor acetylhydrolase, isoform II	Platelet-activating factor acetylhydrolase, isoform II
cluster3_bin.2_NODE_638_length_77192_cov_10.708946_20	BD-FAE	BD-FAE
cluster3_bin.194_NODE_23704_length_11789_cov_7.046787_5	Serine aminopeptidase, S33	Serine aminopeptidase, S33

cluster3_bin.182_NODE_13529_length_16368_cov_7.609820_1	Platelet-activating factor acetylhydrolase, isoform II	Platelet-activating factor acetylhydrolase, isoform II
cluster3_bin.17_NODE_55540_length_6995_cov_6.527522_4	Platelet-activating factor acetylhydrolase, isoform II	Platelet-activating factor acetylhydrolase, isoform II
cluster3_bin.15_NODE_425_length_92888_cov_48.632512_61	Prolyl oligopeptidase family	Prolyl oligopeptidase family
cluster3_bin.139_NODE_65343_length_6334_cov_5.798216_5	BD-FAE	BD-FAE
cluster3_bin.138_NODE_7556_length_22821_cov_16.594176_13	BD-FAE	BD-FAE
cluster3_bin.132_NODE_24470_length_11567_cov_6.324792_3	Cutinase	Cutinase
cluster3_bin.126_NODE_18903_length_13458_cov_4.222786_3	BD-FAE	BD-FAE
cluster3_bin.123_NODE_57883_length_6824_cov_4.432560_8	Serine aminopeptidase, S33	Serine aminopeptidase, S33
cluster3_bin.117_NODE_35894_length_9138_cov_5.377518_8	Platelet-activating factor acetylhydrolase, isoform II	Platelet-activating factor acetylhydrolase, isoform II
cluster3_bin.116_NODE_12699_length_16968_cov_6.442382_5	WD40-like Beta Propeller Repeat	Others
cluster3_bin.114_NODE_6203_length_25440_cov_7.659642_15	Secretion system C-terminal sorting domain	Others
cluster3_bin.103_NODE_1915_length_46442_cov_11.803458_34	Cutinase	Cutinase
cluster3_bin.10_NODE_43327_length_8125_cov_13.005204_6	BD-FAE	BD-FAE
cluster2_bin.97_k127_5800377_2	Dienelactone hydrolase family	Dienelactone hydrolase family
cluster2_bin.82_k127_572997_1	NA	Others
cluster2_bin.66_k127_5083795_49	Serine aminopeptidase, S33	Serine aminopeptidase, S33
cluster2_bin.46_k127_3139108_3	Prolyl oligopeptidase family	Prolyl oligopeptidase family
cluster2_bin.39_k127_3375252_2	Prolyl oligopeptidase family	Prolyl oligopeptidase family
cluster2_bin.296_k127_1768735_36	Carboxylesterase family	Carboxylesterase family
cluster2_bin.27_k127_4808081_2	Prolyl oligopeptidase family	Prolyl oligopeptidase family
cluster2_bin.27_k127_3690957_2	Carboxylesterase family	Carboxylesterase family
cluster2_bin.27_k127_1506491_2	Prolyl oligopeptidase family	Prolyl oligopeptidase family
cluster2_bin.268_k127_811370_1	Secretion system C-terminal sorting domain	Others
cluster2_bin.268_k127_13012_4	Prolyl oligopeptidase family	Prolyl oligopeptidase family
cluster2_bin.263_k127_607476_3	Prolyl oligopeptidase family	Prolyl oligopeptidase family
cluster2_bin.258_k127_5083795_49	Serine aminopeptidase, S33	Serine aminopeptidase, S33
cluster2_bin.257_k127_3139108_3	Prolyl oligopeptidase family	Prolyl oligopeptidase family
cluster2_bin.247_k127_5009065_34	Cutinase	Cutinase
cluster2_bin.237_k127_2403837_4	Alpha/beta hydrolase of unknown function (DUF1400)	Alpha/beta hydrolase family
cluster2_bin.231_k127_60502_45	Secretion system C-terminal sorting domain	Others
cluster2_bin.219_k127_5495289_13	Secretion system C-terminal sorting domain	Others
cluster2_bin.215_k127_2018340_2	Serine aminopeptidase, S33	Serine aminopeptidase, S33
cluster2_bin.2_k127_2403837_4	Alpha/beta hydrolase of unknown function (DUF1400)	Alpha/beta hydrolase family
cluster2_bin.199_k127_2018340_2	Serine aminopeptidase, S33	Serine aminopeptidase, S33

cluster2_bin.197_k127_811370_1	Secretion system C-terminal sorting domain	Others
cluster2_bin.197_k127_13012_4	Prolyl oligopeptidase family	Prolyl oligopeptidase family
cluster2_bin.185_k127_3498345_2	ABC transporter	Others
cluster2_bin.174_k127_4533118_4	BD-FAE	BD-FAE
cluster2_bin.170_k127_3904357_2	Prolyl oligopeptidase family	Prolyl oligopeptidase family
cluster2_bin.167_k127_4808081_2	Prolyl oligopeptidase family	Prolyl oligopeptidase family
cluster2_bin.167_k127_3690957_2	Carboxylesterase family	Carboxylesterase family
cluster2_bin.167_k127_1506491_2	Prolyl oligopeptidase family	Prolyl oligopeptidase family
cluster2_bin.165_k127_60502_45	Secretion system C-terminal sorting domain	Others
cluster2_bin.14_k127_1768735_36	Carboxylesterase family	Carboxylesterase family
cluster2_bin.139_k127_5468278_3	Prolyl oligopeptidase family	Prolyl oligopeptidase family
cluster2_bin.138_k127_5009065_34	Cutinase	Cutinase
cluster2_bin.128_k127_5720365_5	Secretion system C-terminal sorting domain	Others
cluster2_bin.117_k127_607476_3	Prolyl oligopeptidase family	Prolyl oligopeptidase family
cluster2_bin.105_k127_5468278_3	Prolyl oligopeptidase family	Prolyl oligopeptidase family
cluster1_bin.89_k127_1426069_1	Serine aminopeptidase, S33	Serine aminopeptidase, S33
cluster1_bin.87_k127_9630764_289	alpha/beta hydrolase fold	Alpha/beta hydrolase family
cluster1_bin.76_k127_11874394_2	Chlorophyllase	Others
cluster1_bin.70_k127_14074424_1	NA	Others
cluster1_bin.678_k127_2142616_5	Prolyl oligopeptidase family	Prolyl oligopeptidase family
cluster1_bin.677_k127_1433733_1	Serine aminopeptidase, S33	Serine aminopeptidase, S33
cluster1_bin.668_k127_11283838_5	Cutinase	Cutinase
cluster1_bin.66_k127_373155_21	Serine aminopeptidase, S33	Serine aminopeptidase, S33
cluster1_bin.648_k127_1426069_1	Serine aminopeptidase, S33	Serine aminopeptidase, S33
cluster1_bin.637_k127_34855_10	NA	Others
cluster1_bin.630_k127_9571585_3	WD40-like Beta Propeller Repeat	Others
cluster1_bin.60_k127_14074424_1	NA	Others
cluster1_bin.599_k127_8471347_7	Secretion system C-terminal sorting domain	Others
cluster1_bin.582_k127_2383976_5	Chlorophyllase	Others
cluster1_bin.570_k127_11671208_263	Platelet-activating factor acetylhydrolase, isoform II	Platelet-activating factor acetylhydrolase, isoform II
cluster4_bin.99_NODE_14367_length_11491_cov_11.296870_5	NA	Others
cluster4_bin.94_NODE_35946_length_7022_cov_20.449978_4	Prolyl oligopeptidase family	Prolyl oligopeptidase family
cluster4_bin.82_NODE_77256_length_4695_cov_10.951509_2	Serine aminopeptidase, S33	Serine aminopeptidase, S33
cluster4_bin.68_NODE_80554_length_4596_cov_10.551200_4	Serine aminopeptidase, S33	Serine aminopeptidase, S33

cluster4_bin.57_NODE_10247_length_13760_cov_6.942211_4	BD-FAE	BD-FAE
cluster4_bin.51_NODE_7240_length_16441_cov_21.610887_7	BD-FAE	BD-FAE
cluster4_bin.46_NODE_33169_length_7326_cov_6.845826_6	Prolyl oligopeptidase family	Prolyl oligopeptidase family
cluster4_bin.43_NODE_88238_length_4381_cov_8.141008_2	Platelet-activating factor acetylhydrolase, isoform II	Platelet-activating factor acetylhydrolase, isoform II
cluster4_bin.3_NODE_11256_length_13073_cov_20.641112_5	Platelet-activating factor acetylhydrolase, isoform II	Platelet-activating factor acetylhydrolase, isoform II
cluster4_bin.29_NODE_33876_length_7246_cov_4.360033_3	Prolyl oligopeptidase family	Prolyl oligopeptidase family

Supplementary Table S4: Mean RMSF values of putative Antarctic PET hydrolases across triplicate 100 ns molecular dynamics simulations. Highly flexible N- and C-terminal residues were excluded prior to the calculation of means and standard deviations.

Protein	Mean RMSF (Å)	Standard deviations of RMSF (Å)
**Acp1**	0.834	0.589
**Dup1**	0.650	0.557
**Umep1**	4.193	1.673
**Umep2**	0.566	0.230
***Is*PETase**	0.543	0.311

Supplementary Table S5: Root Mean Square Fluctuations of residues surrounding the catalytic residues Ser-His-Asp for each one of the putative PET hydrolases along the molecular dynamics of 100ns. *Is*PETase residue fluctuations are also included. Neighbor residues were considered as all residues at a distance 5 residues around each one of the three catalytic residues in the respective protein sequences.

Protein	Residue	mean_RMSF
Acp1	GLY106	0,57
Acp1	LEU107	0,45
Acp1	LEU108	0,468
Acp1	GLY109	0,441
Acp1	ILE110	0,424
Acp1	SER111	0,505
Acp1	LEU112	0,473
Acp1	GLY113	0,427
Acp1	GLY114	0,459
Acp1	PHE115	0,485
Acp1	LEU116	0,472
Acp1	LEU154	0,451
Acp1	HIS155	0,476
Acp1	GLY156	0,686
Acp1	ASP157	0,891
Acp1	ALA158	1,001
Acp1	ASP159	0,989
Acp1	ARG160	1,187
Acp1	THR161	1,056
Acp1	VAL162	0,896
Acp1	PRO163	1,102
Acp1	VAL164	1,039
Acp1	TYR186	0,669
Acp1	PRO187	0,929
Acp1	GLY188	1,019
Acp1	GLU189	0,785
Acp1	GLY190	0,873
Acp1	HIS 191	0,982
Acp1	MET192	0,979
Acp1	PHE193	0,865
Acp1	HIS 194	1,084
Acp1	GLY195	1,236
Acp1	ASP196	1,164
Dup1	ALA119	0,348
Dup1	VAL120	0,35
Dup1	SER121	0,33
Dup1	GLY122	0,361
Dup1	TRP123	0,366
Dup1	SER124	0,393
Dup1	MET125	0,366
Dup1	GLY126	0,375
Dup1	GLY127	0,382
Dup1	GLY128	0,389
Dup1	GLY129	0,394
Dup1	LEU165	0,4
Dup1	ALA166	0,436
Dup1	GLY167	0,526
Dup1	THR168	0,589
Dup1	ALA169	0,671
Dup1	ASP170	0,607
Dup1	THR171	0,768
Dup1	VAL172	0,688

Dup1	ALA173	0,592
Dup1	PRO174	0,622
Dup1	PRO175	0,648
Dup1	ILE197	0,474
Dup1	GLN198	0,645
Dup1	GLY199	0,785
Dup1	ALA200	0,624
Dup1	ASP201	0,639
Dup1	HIS 202	0,553
Dup1	PHE203	0,658
Dup1	PHE204	0,59
Dup1	PRO205	0,666
Dup1	GLY206	0,943
Dup1	THR207	0,754
Umep1	ALA124	1,38
Umep1	VAL125	1,331
Umep1	ILE126	1,214
Umep1	GLY127	1,74
Umep1	HIS128	2,217
Umep1	SER129	2,874
Umep1	MET130	3,328
Umep1	GLY131	2,719
Umep1	GLY132	2,244
Umep1	GLY133	2,957
Umep1	GLY134	2,918
Umep1	ILE170	2,632
Umep1	ALA171	3,061
Umep1	GLY172	3,595
Umep1	GLU173	4,32
Umep1	GLN174	4,509
Umep1	ASP175	4,027

Umep1	THR176	4,451
Umep1	THR177	4,154
Umep1	VAL178	3,57
Umep1	THR179	3,888
Umep1	PRO180	3,892
Umep1	LEU201	4,008
Umep1	THR202	4,786
Umep1	GLY203	4,933
Umep1	ALA204	4,175
Umep1	GLY205	3,99
Umep1	HIS 206	3,484
Umep1	GLY207	3,452
Umep1	PHE208	3,299
Umep1	PRO209	2,816
Umep1	SER210	3,22
Umep1	ARG211	3,895
Umep2	ALA123	0,305
Umep2	VAL124	0,299
Umep2	MET125	0,325
Umep2	GLY126	0,397
Umep2	HIS 127	0,425
Umep2	SER128	0,434
Umep2	MET129	0,432
Umep2	GLY130	0,404
Umep2	GLY131	0,426
Umep2	GLY132	0,463
Umep2	GLY133	0,419
Umep2	LEU169	0,367
Umep2	ALA170	0,439
Umep2	GLY171	0,543
Umep2	GLN172	0,692

Umep2	ARG173	0,831
Umep2	ASP174	0,874
Umep2	GLY175	1,274
Umep2	THR176	1,14
Umep2	VAL177	0,699
Umep2	SER178	0,685
Umep2	PRO179	0,598
Umep2	LEU200	0,475
Umep2	THR201	0,615
Umep2	GLY202	0,798
Umep2	ALA203	0,646
Umep2	ALA204	0,807
Umep2	HIS 205	0,876
Umep2	SER206	0,934
Umep2	PHE207	0,661
Umep2	PRO208	0,716
Umep2	THR209	0,676
Umep2	SER210	0,734
*Is*PETase	GLY155	0,34
*Is*PETase	VAL156	0,292
*Is*PETase	MET157	0,315
*Is*PETase	GLY158	0,345
*Is*PETase	TRP159	0,336
*Is*PETase	SER160	0,404
*Is*PETase	MET161	0,4
*Is*PETase	GLY162	0,385
*Is*PETase	GLY163	0,379
*Is*PETase	GLY164	0,417
*Is*PETase	GLY165	0,385
*Is*PETase	PHE201	0,348
*Is*PETase	ALA202	0,395

*Is*PETase	CYS 203	0,45
*Is*PETase	GLU204	0,677
*Is*PETase	ASN205	1,041
*Is*PETase	ASP206	1,211
*Is*PETase	SER207	1,824
*Is*PETase	ILE208	1,782
*Is*PETase	ALA209	1,513
*Is*PETase	PRO210	1,081
*Is*PETase	VAL211	0,622
*Is*PETase	ILE232	0,468
*Is*PETase	ASN233	0,683
*Is*PETase	GLY234	1,26
*Is*PETase	GLY235	1,702
*Is*PETase	SER236	1,298
*Is*PETase	HIS237	1,061
*Is*PETase	SER238	0,557
*Is*PETase	CYS 239	0,465
*Is*PETase	ALA240	0,466
*Is*PETase	ASN241	0,451
*Is*PETase	SER242	0,532

Supplementary Table S6: Gibbs free energies predicted from molecular docking analysis of the four candidate pPETHs with 2PET (ethylene glycol terephthalate (3:2)) as ligand according to Vina empirical scoring function.

Protein	Gibbs Free Energy (Kcal//mol)
**Umep1**	**-6.1**
**Umep2**	**-6.1**
**Acp1**	**-7.4**
**Dup1**	**-4.7**

Supplementary Table S7: Protein-ligand interactions inferred for putative PET hydrolases bound to the 2PET ligand dimer (ethylene glycol terephthalate (3:2)). The 2PET ligand is designated by the code "UNK1", and the "Name" column specifies the exact interacting atoms for each contact. Distances are provided in angstroms.

Protein	Name	Distance	Types	Category
Acp1	:SER34:HN - A:UNK1:O8	2,31386	Conventional Hydrogen Bond	Hydrogen Bond
Acp1	:ASN35:HN - A:UNK1:O10	2,32703	Conventional Hydrogen Bond	Hydrogen Bond
Acp1	:LEU112:HN - A:UNK1:O8	1,82671	Conventional Hydrogen Bond	Hydrogen Bond
Acp1	A:UNK1:H22 - :SER34:OG	1,97732	Conventional Hydrogen Bond	Hydrogen Bond
Acp1	:GLY33:HA1 - A:UNK1:O8	2,97953	Carbon Hydrogen Bond	Hydrogen Bond
Acp1	:SER111:HB2 - A:UNK1:O8	2,42396	Carbon Hydrogen Bond	Hydrogen Bond
Acp1	A:UNK1:C5 - :THR161:O	3,31376	Carbon Hydrogen Bond	Hydrogen Bond
Acp1	:PHE115 - A:UNK1	3,88478	π - π Stacked	Hydrophobic
Acp1	:TYR73 - A:UNK1	5,48547	π - π Stacked	Hydrophobic
Acp1	A:UNK1 - :LEU112	4,99946	π -Alkyl	Hydrophobic
Dup1	:PHE54:HN - A:UNK1:O6	2,37296	Conventional Hydrogen Bond	Hydrogen Bond

Dup1	:PHE203:HN - A:UNK1:O3	2,17934	Conventional Hydrogen Bond	Hydrogen Bond
Dup1	:PHE203:HN - A:UNK1:O9	2,22888	Conventional Hydrogen Bond	Hydrogen Bond
Dup1	A:UNK1:H22 - A:UNK1:O8	2,63135	Conventional Hydrogen Bond	Hydrogen Bond
Dup1	:GLY53:HA1 - A:UNK1:O6	2,88496	Carbon Hydrogen Bond	Hydrogen Bond
Dup1	:PHE203:HB2 - A:UNK1	2,44113	π -Sigma	Hydrophobic
Dup1	A:UNK1 - :MET125	5,03984	π Alkyl	Hydrophobic
Dup1	A:UNK1 - :VAL172	5,33156	π -Alkyl	Hydrophobic
Umep1	:TYR61:HN - :UNK1:O5	2,42018	Conventional Hydrogen Bond	Hydrogen Bond
Umep1	:THR64:HN - :UNK1:O7	2,04613	Conventional Hydrogen Bond	Hydrogen Bond
Umep1	:GLY63:HA1 - :UNK1:O7	2,87026	Carbon Hydrogen Bond	Hydrogen Bond
Umep1	:PHE156 - :UNK1	4,9468	π - π T-shaped	Hydrophobic
Umep1	:UNK1 - :ALA154	4,92983	π -Alkyl	Hydrophobic
Umep2	:SER154:HN - A:UNK1:O7	2,8923	Conventional Hydrogen Bond	Hydrogen Bond
Umep2	:ALA155:HN - A:UNK1:O7	2,1427	Conventional Hydrogen Bond	Hydrogen Bond
Umep2	A:UNK1:H22 - :GLU66:OE1	2,51074	Conventional Hydrogen Bond	Hydrogen Bond
Umep2	:GLY58:HA2 - A:UNK1:O8	2,47997	Carbon Hydrogen Bond	Hydrogen Bond
Umep2	:HIS127:HE1 - A:UNK1:O8	2,87476	Carbon Hydrogen Bond	Hydrogen Bond
Umep2	:TYR153:HA - A:UNK1:O7	2,33515	Carbon Hydrogen Bond	Hydrogen Bond
Umep2	:TYR153 - A:UNK1	3,84023	π - π Stacked	Hydrophobic
